# Nanoscale Metal–Organic Frameworks‐Mediated Degradation of Mutant p53 Proteins and Activation of cGAS‐STING Pathway for Enhanced Cancer Immunotherapy

**DOI:** 10.1002/advs.202307278

**Published:** 2024-01-15

**Authors:** Li Sun, Hongbo Gao, Han Wang, Jingwei Zhou, Xiuru Ji, Yuxin Jiao, Xiaojia Qin, Dalong Ni, Xiangpeng Zheng

**Affiliations:** ^1^ Department of Radiation Oncology Huadong Hospital Fudan University Shanghai 200040 P. R. China; ^2^ Department of Orthopaedics Shanghai Key Laboratory for Prevention and Treatment of Bone and Joint Diseases Shanghai Institute of Traumatology and Orthopaedics Ruijin Hospital Shanghai Jiao Tong University School of Medicine Shanghai 200025 P. R. China; ^3^ Department of Plastic and Reconstructive Surgery Shanghai Ninth People's Hospital Shanghai Jiao Tong University School of Medicine Shanghai 200011 P. R. China

**Keywords:** aPD‐L1, cGAS‐STING, immunotherapy, metal–organic frameworks, mutant p53, proteasomal degradation

## Abstract

Activating cGAS‐STING pathway has great potential to achieve effective antitumor immunotherapy. However, mutant p53 (mutp53), a commonly observed genetic alteration in over 50% of human cancer, will impede the therapeutic performance of the cGAS‐STING pathway. Herein, multifunctional ZIF‐8@MnO_2_ nanoparticles are constructed to degrade mutp53 and facilitate the cGAS‐STING pathway. The synthesized ZIF‐8@MnO_2_ can release Zn^2+^ and Mn^2+^ in cancer cells to induce oxidative stress and cytoplasmic leakage of fragmented mitochondrial double‐stranded DNAs (dsDNAs). Importantly, the released Zn^2+^ induces variable degradation of multifarious p53 mutants through proteasome ubiquitination, which can alleviate the inhibitory effects of mutp53 on the cGAS‐STING pathway. In addition, the released Mn^2+^ further increases the sensitivity of cGAS to dsDNAs as immunostimulatory signals. Both in vitro and in vivo results demonstrate that ZIF‐8@MnO_2_ effectively promotes the cGAS‐STING pathway and synergizes with PD‐L1 checkpoint blockades, leading to remarkable regression of local tumors as well as distant metastases of breast cancer. This study proposes an inorganic metal ion‐based nanoplatform to enhance the cGAS‐STING‐mediated antitumor immunotherapy, especially to those tumors with mutp53 expression.

## Graphical abstract

A nanotechnology‐based Mn–Zn dual‐ion delivery platform is constructed. It can enhance the cGAS‐STING‐mediated anti‐tumor immunotherapy via. multiple aspects, including GSH responsive, cGAS enzymatic activity‐modulated, and mutp53‐targeted manifold pathways. This study lays a foundation for designing therapeutic strategies targeting the cGAS‐STING pathway and offers new immunotherapeutic options for patients with overexpressed mutant p53.

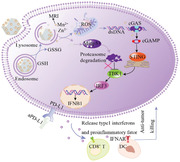

## 1 Introduction

Immunotherapy has emerged as a revolutionary milestone in cancer treatment.^[^ ^1^ ^]^ Immune checkpoint inhibitors (ICIs), adoptive cell transfer, and cancer vaccines have shown encouraging results in various malignancies. However, because of the immunosuppressive tumor microenvironments, only a limited portion of cancer patients truly benefit from the immunotherapy.^[^ ^2^ ^]^ Recent studies have shown that the mutant p53 (mutp53), the commonly observed genetic alterations in over 50% of human cancer,^[^ ^3^ ^]^ could make contributions to immunosuppressive microenvironments by inhibiting the cGAS‐STING pathway. Generally, during the process of cGAS‐STING pathway activation, once the cytoplasmic double‐stranded DNAs (dsDNAs) are sensed by cyclic GMP‐AMP synthase (cGAS), it catalyzes the production of cyclic GMP‐AMP (cGAMP), a second messenger molecule that binds to the stimulator of interferon genes (STING). Subsequently, a signaling cascade leads to the production of type I interferons (IFNs) and pro‐inflammatory cytokines against pathogens and cancer cells.^[^ ^4^ ^]^ However, mutp53 can inhibit the cGAS‐STING pathway by interacting with TANK‐binding kinase 1 (TBK1) and preventing the formation of trimeric complex between TBK1, STING, and IRF3, which is required for the activation of IRF3. Consequently, this interference reduces the sensitivity of cGAS to cytoplasmic DNAs and further suppresses the activation of innate immunity.^[^ ^5^ ^]^ Hence, for achieving efficient immunotherapy, it is important to activate the cGAS‐STING pathway by inhibiting mutp53 simultaneously.

Inorganic metal ions, such as Na^+^, K^+^, Zn^2+^, Mg^2+^, and Mn^2+^ play important roles in regulating cell physiological activities.^[^ ^6^ ^]^ For example, the binding of a single Zn^2+^ is required for proper folding of the p53 protein. Since mutp53 has a weakened binding capability with Zn^2+^, excess Zn^2+^ will induce the degradation of mutp53, which benefits the recovery of the cGAS‐STING pathway.^[^ ^7^ ^]^ Meanwhile, Mn^2+^ can directly bind to cGAS and enhance its sensitivity to dsDNAs and enzymatic activity, which will promote the activation of the cGAS‐STING signaling.^[^ ^8^ ^]^ Furthermore, Zn^2+^ and Mn^2+^ can both induce oxidative stress in cancer cells and damage the mitochondria, which will induce the release of mitochondrial dsDNA (mtDNA) and trigger the cGAS‐STING signaling pathway.^[^ ^9^ ^]^ Hence, simultaneously delivering Zn^2+^ and Mn^2+^ to cancer cells has much potential to enhance the effect of the cGAS‐STING pathway.

**Figure 1:**
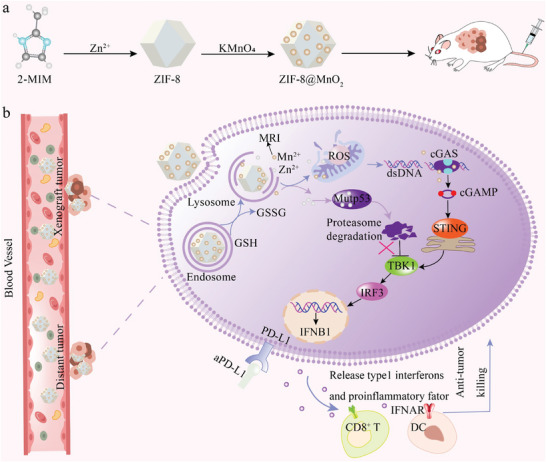
a) Schematic illustration of the synthetic route to ZIF‐8@MnO_2_; b) mechanism of degrading broad‐spectrum mutp53 proteins for activation of cGAS‐STING pathway.

Metal–organic frameworks (MOFs), a class of materials consisting of metal ions with organic ligands, exhibit many enviable properties including good biocompatibility, rich chemical diversity, and high surface area.^[^ ^10^ ^]^ Nanoscale MOFs have been widely used in drug delivery, photodynamic therapy, and radiation therapy for cancer.^[^ ^11^ ^]^ Herein, we constructed MnO_2_‐modified zeolitic imidazolate framework‐8 (ZIF‐8, a kind of Zn^2+^ based MOF) nanoparticles to degrade mutp53 and activate the cGAS‐STING pathway for enhanced immunotherapy. As illustrated in **Figure** **1**, due to the highly expressed glutathione (GSH) and low pH in the tumor, ZIF‐8@MnO_2_ is decomposed to release Mn^2+^ and Zn^2+^ ions. As described above, Mn^2+^ and Zn^2+^ could activate the cGAS‐STING pathway via. multiple aspects. To validate the applicability and generality of Zn^2+^ induced degradation of mutp53, in vitro experiments in four kinds of breast cancer cell lines with distinct mutations were used, including MCF7 (WT), MDA‐MB231 (p53R280K), BT549 (p53R249S), and SKBR3 (p53R175H). It is found that certain cytoplasmic mutp53 proteins could be eliminated through proteasome degradation, resulting in the recovery of STING‐TBK1‐IRF3 trimeric complex and decreased inhibitory effects of mutp53 on the cGAS‐STING pathway. In vivo experiments with 4T1 (p53R249S) allografts further verified in vitro results, and the synergizing effects between ZIF‐8@MnO_2_ and PD‐L1 checkpoint inhibitors were also demonstrated. Overall, this study presented promising methods to enhance the cGAS‐STING‐mediated immunotherapy by using dual metal ions regulation in single nanoplatforms, especially for those tumors with mutp53 expression.

## 2 Results and Discussion

### 2.1 Synthesis and Characterization of ZIF‐8@MnO_2_

**Figure 2:**
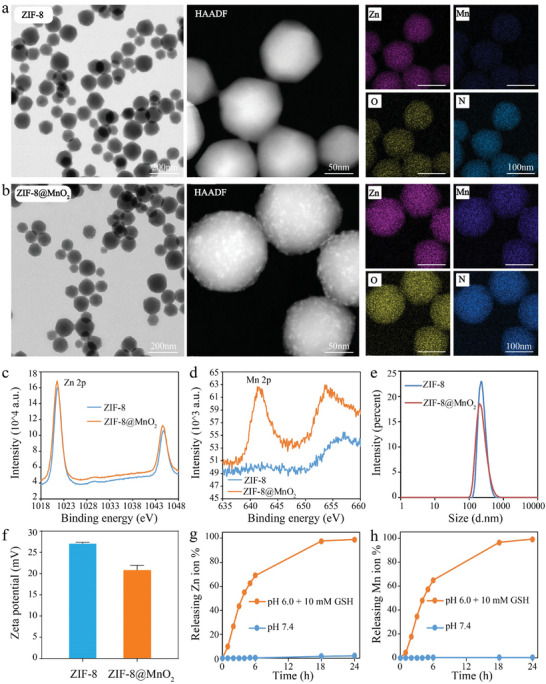
Characterization of ZIF‐8@MnO_2_. a) SEM, TEM, and element mapping images of ZIF‐8 nanoparticles. b) SEM, TEM, and element mapping images of ZIF‐8@MnO_2_. XPS spectra: c) ZIF‐8; d) ZIF‐8@MnO_2_. e) DLS analysis of ZIF‐8 and ZIF‐8@MnO_2_. f) Zeta potential distributions of ZIF‐8 and ZIF‐8@MnO_2_. g) Zn^2+^ release over time at different pH values. h) Mn^2+^release over time at different pH values.

First, ZIF‐8 nanoparticles were prepared using previously reported method.^[^ ^12^ ^]^ Subsequently, a thin layer of MnO_2_ was grown on the surface of ZIF‐8 nanoparticles. Transmission electron microscopy (TEM) and high‐angle annular dark‐field (HAADF) imaging showed that the synthesized ZIF‐8 and ZIF‐8@MnO_2_ nanoparticles had similar sizes, but the surface of ZIF‐8 was smooth while ZIF‐8@MnO_2_ exhibited a rough surface because of the MnO_2_ modification. Elemental mapping indicated that ZIF‐8 contained Zn elements, and ZIF‐8@MnO_2_ contained both Zn and Mn elements (**Figure** **2**a,b), which were in accordance with the data of X‐ray photoelectron spectroscopy (XPS) (Figure 2c,d). Individual MnO_2_ nanoparticles were also synthesized as a control, which was shown by TEM image in Figure S1 (Supporting Information). The hydrodynamic diameter of ZIF‐8@MnO_2_ was about 248.3 nm (Figure 2e), slightly larger than ZIF‐8 (241.2 nm). Zeta potential of ZIF‐8@MnO_2_ was slightly less than ZIF‐8 (Figure 2f). Fourier transform infrared spectroscopy (FTIR) (Figure S2, Supporting Information) and ultraviolet–visible (UV–vis) spectroscopy (Figure S3, Supporting Information) of ZIF‐8@MnO_2_ and ZIF‐8 showed few differences. Nonsignificant differences were detected in terms of hydrodynamic size (Figure S4, Supporting Information) on the 1, 7, and 14th day in physiological solution, respectively. These data presented the successful synthesis of ZIF‐8@MnO_2_ nanoparticles.

It has been reported that acidic environment could lead to the degradation of ZIF‐8, while the combination of acidic environment and glutathione (GSH) could lead to the degradation of MnO_2_.^[^ ^13^ ^]^ As cancer cells often exhibit subacidity (lysosomes) and overexpressed GSH, the ZIF‐8@MnO_2_ would be degraded to release Zn^2+^ and Mn^2+^ in cancer cells after cytophagy. To prove this point, the release of Zn^2+^ and Mn^2+^ were measured in simulated fluid (Figure 2g,h). The result showed that Zn^2+^ and Mn^2+^ could be completely released in 24 h in an acidic PBS (pH 6.0, GSH 10 mM), while they could not be released in neutral PBS (pH 7.4, no GSH). In addition, the released Mn^2+^ could present a T_1_‐weighted magnetic resonance imaging (MRI) property (Figure S5a,b, Supporting Information).^[^ ^14^ ^]^ Hence, ZIF‐8@MnO_2_ could be used for cancer microenvironment responsive MRI, which benefits the precise imaging during therapy. In vivo experiments showed that, when ZIF‐8@MnO_2_ was intravenously administrated to tumor‐bearing mice via. the tail vein, tumor area was enhanced in MRI and the maximum T_1_ signal was observed at 12 h (Figure S5c, Supporting Information).

### 2.2 Performance and Cytotoxicity Study of ZIF‐8@MnO_2_ In Vitro

**Figure 3:**
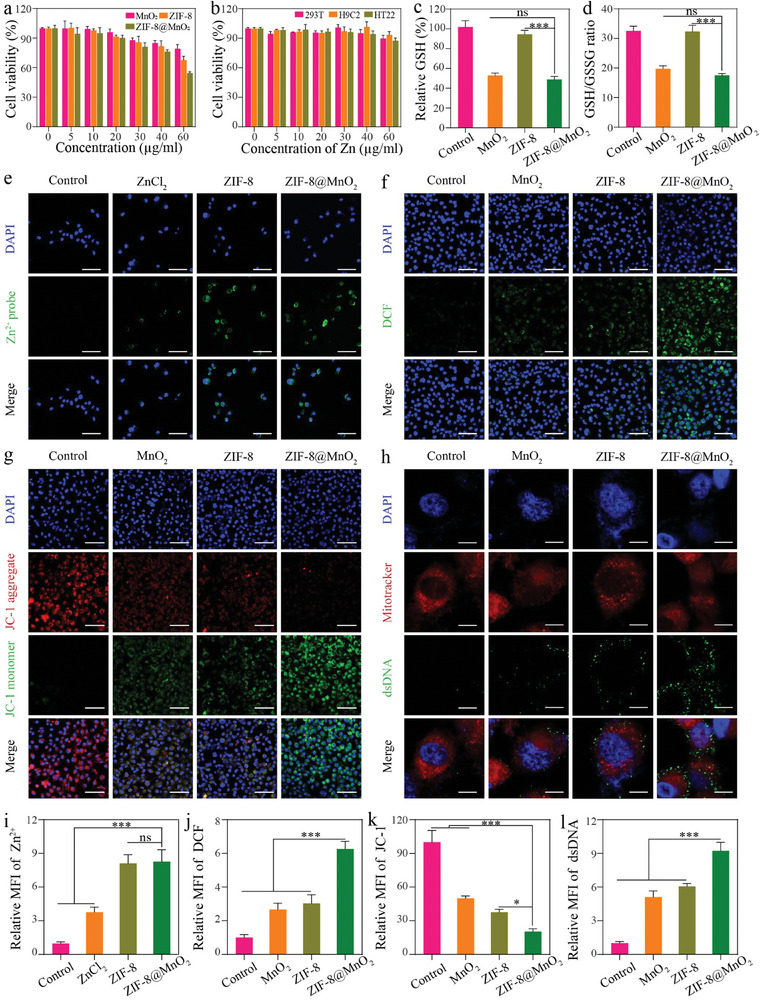
Performance and cytotoxicity study of ZIF‐8@MnO_2_ nanoparticles in vitro. a) Cell viability of BT549 cells after various treatments with PBS, MnO_2_, ZIF‐8, and ZIF‐8@MnO_2_; b) cytotoxicity of ZIF‐8@MnO_2_ in 293T, H9C2, and HT22 cells (*n* = 3, mean ± SD). c) Relative amount of GSH in BT549 cells after treatment compared to control (*n* = 3, mean ± SD). d) GSH/GSSG ratio in BT549 cells after treatment (*n* = 3, mean ± SD). e) Fluorescence image of Metal Fluor™ Zn‐520 (green) stained BT549 cells after incubation with ZnCl_2_, ZIF‐8, and ZIF‐8@MnO_2_ nanoparticles for 8 h. Scar bar: 100 µm. f) Fluorescence images of BT549 cells with ROS produced after different treatments (PBS, MnO_2_, ZIF‐8, and ZIF‐8@MnO_2_). Scar bar: 100 µm. g) Alterations in mitochondrial membrane potential in BT549 cells. Scar bar: 100 µm. h) Detection of cytoplasmic dsDNA in BT549 cells after different treatments (PBS, MnO_2_, ZIF‐8, and ZIF‐8@MnO_2_). Scar bar: 20 µm. i) Statistical analysis of immuno‐fluorescence staining of Zn^2+^. j) Statistical analysis of ROS. k) Statistical analysis of JC‐1 (Red/Green). l) Statistical analysis of dsDNA. One‐way analysis of variance (ANOVA) was performed (i–l): ***p < 0.001.

To evaluate in vitro cytotoxicity of ZIF‐8@MnO_2_, the CCK‐8 assay was conducted using breast cancer cell line BT549. The results showed that ZIF‐8@MnO_2_ nanoparticles exhibited a concentration‐dependent effect on tumor cell viability, while no significant effect on the viability of three normal cell lines was observed at the same concentration (**Figure** **3**a,b). It has been reported that MnO_2_ could consume GSH and the released Mn^2+^ and Zn^2+^ could induce the generation of ROS.^[^ ^15^ ^]^ To investigate the effect of ZIF‐8@MnO_2_ on cellular redox homeostasis, the reduced GSH level and the enhanced ROS level were evaluated. It was observed that the total GSH content and the GSH/GSSG ratio in cells were obviously decreased by ZIF‐8@MnO_2_ (Figure 3c,d). To measure the intracellular concentration of Zn^2+^, immuno‐fluorescence examination was conducted using Metal Fluor Zn‐520. Those cells treated with ZIF‐8@MnO_2_ or ZIF‐8 nanoparticles exhibited stronger fluorescence intensity than those treated with the equivalent concentration of ZnCl_2_, indicating that these nanoparticles could enter into cells through cytophagy (Figure 3e,i), the single cell inductively coupled plasma mass spectrometry (SC–ICP–MS) analysis presented the same trend in Figure S6 (Supporting Information). The DCFH‐DA assay kit was applied to assess the intracellular ROS (Figure 3f,j). Confocal laser scanning microscopy (CLSM) imaging revealed that treatment with ZIF‐8@MnO_2_ significantly increased intracellular ROS levels. The mitochondrial membrane potential was impaired due to ROS‐induced oxidative stress, which was manifested as a decrease in JC‐1 aggregates and an increase in JC‐1 monomers (Figure 3g,k). The injured mitochondria will release dsDNAs into the cytoplasm to trigger the cGAS‐STING pathway.^[^ ^16^ ^]^ As shown by mitotracker staining of mitochondria, mitochondrial integrity was damaged, which could promote the release of the mtDNA into the cytoplasm (Figure 3h,l).

### 2.3 ZIF‐8@MnO_2_ Promotes cGAS‐STING Pathway

**Figure 4:**
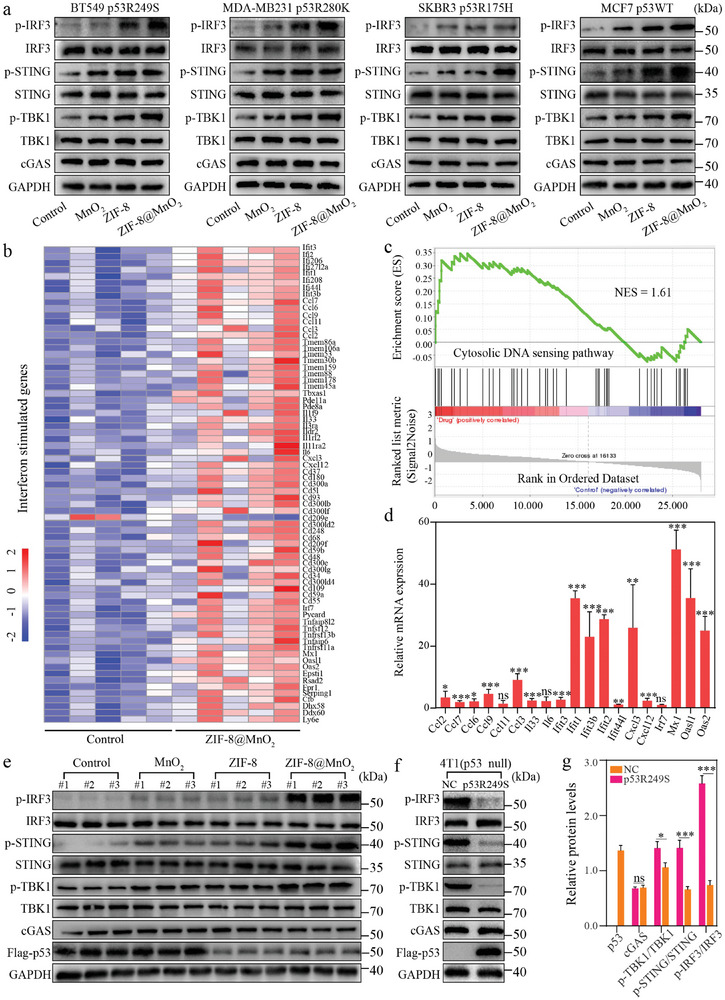
cGAS‐STING pathway activation after the treatment of ZIF‐8@MnO_2_. a) Activation of cGAS‐STING pathway in cells (BT549, MDA‐MB231, SKBR3, MCF7) after ZIF‐8@MnO_2_ treatment. b) Heatmap of RNA‐seq for the transcription level analysis of ISGs in mice with or without treatment of ZIF‐8@MnO_2_ (*n* = 5). c) Gene set enrichment analysis (GSEA) of Reactome pathway (i.e., DNA‐sensing signaling pathway). d) RT‐qPCR analysis of ISGs (*n* = 3, mean ± SD). e) Western blotting analysis of cGAS, p53, TBK1, p‐TBK1, STING, p‐STING, IRF3, and p‐IRF3 protein levels in randomly selected tumor samples. f) Western blotting analysis; g) statistical analysis of the pathway of 4T1 (NC group was the negative control group) and 4T1 (p53R249S). Student's *t*‐test (d,g) was performed: *p < 0.05, **p < 0.01, ***p < 0.001.

Having proved that ZIF‐8@MnO_2_ could release Zn^2+^ and Mn^2+^ to facilitate the release of mitochondria dsDNA, the effect of ZIF‐8@MnO_2_ on the cGAS‐STING pathway was comprehensively investigated. First, qPCR with reverse transcription (RT‐qPCR) experiments were performed to evaluate the expression of downstream target genes IFNB1 and ISG56 in BT549, SKBR3, and MCF7 cells, respectively, while target genes CXCL10 and ISG56 in MDA‐MB231 cells were also measured (Figure S7, Supporting Information). Activation of the cGAS‐STING pathway promotes IFNB1 transcription to encode IFNs, which is a vital immune protein that modulates the expression of other immune‐related genes, such as ISG56 and CXCL10. These regulatory mechanisms determine the biological outcome of tumor killing effect by IFNs response, which are important for maintaining immune homeostasis and exerting anti‐tumor immunity.^[^ ^17^ ^]^ Clearly, the expression of these genes and the phosphorylation levels of pathway‐related proteins (TBK1, STING, IRF3) were increased in ZIF‐8@MnO_2_ treated cells (**Figure** **4**a; Figure S8, Supporting Information). These findings represented up‐regulated immunology pathways and supported that the ZIF‐8@MnO_2_ nanoparticles could effectively activate the immune response through the cGAS‐STING pathway in vitro.

As mentioned earlier, the mutp53 protein could inhibit the activation of cGAS‐STING pathway by interfering with TBK1, rendering innate immunity inactive or inert.^[^ ^5^ ^]^ In this regard, we introduced exogenous mutp53 protein by constructing 4T1 (p53R249S) overexpression cells (Figure S9, Supporting Information). In vivo models of 4T1 (p53R249S) tumor‐bearing mice were used to validate the impact of ZIF‐8@MnO_2_ on the activity of the cGAS‐STING pathway. Tissue RNA was extracted for RNA sequencing (RNA‐seq) analysis to study the gene expression profile of 4T1 tumors after ZIF‐8@MnO_2_ treatment. During the analysis, 994 differentially expressed genes were identified (Figure S10, Supporting Information). Gene ontology (GO) enrichment analysis (Figure S11, Supporting Information) revealed significant enrichment of immune‐related pathways in the ZIF‐8@MnO_2_ treated tumors, especially including the DNA sensing signaling pathway and induction of interferon‐stimulated genes (ISGs) expression (Figure 4b,c). The ISGs are a group of downstream target genes of cGAS‐STING pathway that are induced by the stimulation of IFNs as activated by cGAS‐STING pathway. Therefore, the up‐regulation of ISGs expression is compelling evidence of the activation and functionality of the cGAS‐STING pathway. In detail, when cells are exposed to IFNs, specific signaling pathways are activated, resulting in the nuclear translocation of the transcription factor STAT and its binding to IFN‐stimulated response elements (ISREs), thereby promoting the transcription of ISGs. Then, the ISGs can encode various proteins with diverse functions, such as anti‐tumor proteins and immune regulatory factors, which contribute to tumor suppression and participation in immune responses.^[^ ^18^ ^]^ The expression of representative nineteen ISGs were all measured by RT‐qPCR, and the expression trends were in accordance with the RNA‐seq analysis results (Figure 4d).

Subsequently, three tumor samples were randomly selected from each group, and the protein was extracted to measure the phosphorylation levels of TBK1, STING, and IRF3, which were increased in activated cGAS‐STING pathways. The results proved that treatment with ZIF‐8@MnO_2_ could effectively activate the cGAS‐STING signaling pathway in the animal model, which was consistent with the findings of in vitro experiments (Figure 4e; Figure S12, Supporting Information). In addition, due to the expression of mutp53, the component proteins of the trimeric complex in the cGAS‐STING pathway were significantly down‐regulated compared with the negative control group (NC) as shown in Figure 4f,g, which was in agreement with previous studies. Therefore, we performed additional verification to determine whether the activation of cGAS‐STING pathway is associated with the reduction of mutp53.

### 2.4 ZIF‐8@MnO_2_ Can Induce the Degradation of Mutp53 Proteins

**Figure 5:**
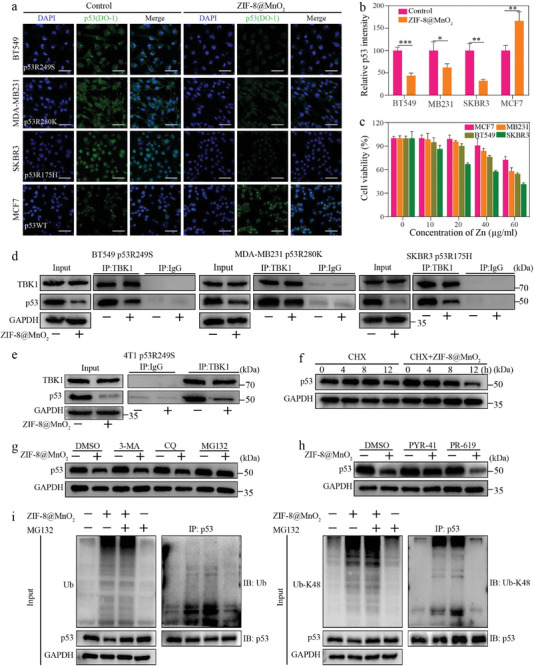
Mechanism of degradation of mutp53 proteins. a) Effect of ZIF‐8@MnO_2_ treatment on p53 proteins content in four breast cancer cell lines (BT549, MDA‐MB231, SKBR3, and MCF7). Scar bar: 100 µm. b) Statistical analysis of p53 protein before and after treatment (*n* = 3, mean ± SD). c) Cytotoxicity of ZIF‐8@MnO_2_ in cells with different states of p53 protein (*n* = 3, mean ± SD). d) The interaction between TBK1 and p53 in breast cancer cells after ZIF‐8@MnO_2_ treatment. e) The interaction between TBK1 and exogenous mutp53 protein after ZIF‐8@MnO_2_ treatment. f) Western blotting of p53 in BT549 cells treated with cycloheximide (CHX) with or without ZIF‐8@MnO_2_. g) Western blotting of p53 in BT549 cells treated with autophagy inhibitors 3‐methyladenine (3MA), chloroquine diphosphate salt (CQ), and the 26S proteasome inhibitor MG132 with or without ZIF‐8@MnO_2_. Dimethyl sulphoxide (DMSO) worked as a negative control. h) Western blotting of p53 in BT549 cells treated with E1 enzyme inhibitor PYR‐41 and the deubiquitinase (DUB) inhibitor PR‐619 with or without ZIF‐8@MnO_2_. i) Coimmunoprecipitation analysis of total and K48‐linked ubiquitination of immunoprecipitated p53 in BT549 cells with the treatment of ZIF‐8@MnO_2_. Student's *t*‐test (b) was performed: *p < 0.05, **p < 0.01.

The majority (over 70%) of p53 mutations involve the substitution of a single amino acid within its DNA‐binding domain (DBD). These mutations can be broadly classified into two categories: contact mutations (e.g., R273H, R280K) and conformational mutations (e.g., R175H, R249S, Y220C). They can lead to structural changes in the p53 protein, resulting in the loss of tumor suppression and thus promoting tumorigenesis and progression via. diverse mechanisms.^[^ ^19^ ^]^ Besides the impact on the cGAS‐STING pathway, it is necessary to validate the effect of ZIF‐8@MnO_2_ on mutated and wild‐type p53 proteins. Four breast cancer cell lines were verified with mutation status prior to experiments. Among them, BT549 (p53R249S) and SKBR3 (p53R175H) were conformational mutations, MDA‐MB231 (p53R280K) was a contact mutation, and MCF7 was wild‐type. After treatment with ZIF‐8@MnO_2_, three p53 mutated cell lines showed remarkably weakened p53 fluorescence intensity, while the protein content of p53 in the wild‐type cell line was increased, implying that ZIF‐8@MnO_2_ had differential effects on mutated and wild‐type p53 proteins with a tendency of selectively promoting mutp53 protein degradation (**Figure** **5**a,b). In addition, the accumulation of mutp53 proteins is correlated with increased proliferation of tumor cells.^[^ ^20^ ^]^ The cell viability experiments showed a significantly decreased viability of ZIF‐8@MnO_2_ treated p53‐mutated tumor cells compared to that of wild‐type conformation, which was attributed to the decreased mutp53 as degraded by ZIF‐8@MnO_2_ (Figure 5c).

Notably, the coimmunoprecipitation experiments demonstrated that alterations in the interaction between TBK1 and p53 were associated with the decrease of mutp53 proteins (Figure 5d; Figure S13a,b, Supporting Information). In addition, the impact of ZIF‐8@MnO_2_ on exogenous mutp53 proteins was explored, and we found that ZIF‐8@MnO_2_ could also attenuate the interaction between TBK1 and mutp53, which verified in vivo results (Figure 5e; Figure S13c, Supporting Information). Furthermore, underlying mechanisms of nanoparticle‐induced mutp53 reduction were investigated. As known, intracellular protein degradation mainly depends on two systems‐autophagy and proteasome ubiquitination.^[^ ^21^ ^]^ BT549 cells were treated with the protein synthesis inhibitor cycloheximide (CHX) in combination with ZIF‐8@MnO_2_. The immunoblotting showed an accelerated decrease in mutp53 levels compared to the control group (Figure 5f; Figure S13d, Supporting Information). In control, cells were treated with ZIF‐8@MnO_2_ alone or in combination with the autophagy inhibitors, 3‐methyladenine (3MA) and chloroquine diphosphate salt (CQ), as well as the 26S proteasome inhibitor MG132. The results showed that 3MA and CQ treatment failed to inhibit the decrease of mutp53 protein levels, while MG132 treatment maintained the mutp53 levels regardless of the administration of ZIF‐8@MnO_2_ (Figure 5g; Figure S13e, Supporting Information), which indicated a proteasome ubiquitination pathway. Furthermore, E1 enzyme inhibitor PYR‐41 and deubiquitinase (DUB) inhibitor PR‐619 were used to inhibit or promote the ubiquitination process, which showed opposite responses of mutp53 protein levels (Figure 5h; Figure S13f, Supporting Information). Consistently, the coimmunoprecipitation experiments showed that the ubiquitination level of mutp53, especially the K48 polyubiquitination level that related to protein degradation, was elevated after ZIF‐8@MnO_2_ treatment (Figure 5i). Taken together, these findings indicated that ZIF‐8@MnO_2_ can facilitate mutp53 protein degradation through the ubiquitination‐dependent proteasome pathway.

### 2.5 The Therapeutic Effect of ZIF‐8@MnO_2_ In Vitro

First, the mechanism of ZIF‐8@MnO_2_‐mediated cell death was investigated. As previously reported, the Bax protein can interact with IRF3 and translocate to mitochondria, leading to the release of Cyto‐C and promoting cell apoptosis.^[^ ^22^ ^]^ After co‐incubating BT549 cells with ZIF‐8@MnO_2_, the expression of apoptosis‐related proteins, such as BAX, Cyto‐C, cleaved caspase‐3, and cleaved caspase‐9 were increased as shown by immunoblotting (Figure S14, Supporting Information), which demonstrated that ZIF‐8@MnO_2_ could enhance the expression of apoptosis‐related proteins. Cellular apoptosis was also assessed by membrane‐associated protein Annexin V‐FITC staining. Compared to MnO_2_ (4.87%) and ZIF‐8 (6.35%)‐treated groups, ZIF‐8@MnO_2_ treatment induced more pronounced cell apoptosis (13.24%) (Figure S15, Supporting Information), suggesting that ZIF‐8@MnO_2_ promoted cellular death through the apoptotic pathway. Subsequently, classical scratch experiments and Transwell assays were used to evaluate the effect of ZIF‐8@MnO_2_ on tumor cell migration and invasion. It was found that ZIF‐8@MnO_2_ nanoparticles significantly inhibited the migration capability of BT549 cells in comparison to the PBS‐treated group (Figure S16, Supporting Information). Similar results were also observed in the Transwell assay (Figure S17, Supporting Information). These findings indicated that ZIF‐8@MnO_2_ impaired the migration and invasion of breast cancer cells.

### 2.6 Biocompatibility and Therapeutic Efficiency of ZIF‐8@MnO_2_ Nanoparticles In Vivo

We first evaluated the biocompatibility of ZIF‐8@MnO_2_ nanoparticles. BALB/c mice were injected with nanoparticles via. the tail vein, and hematological and biochemical tests were performed on the 15th day after injection (Figure S18, Supporting Information). Additionally, major organs, including the heart, liver, spleen, lungs, and kidneys were collected for histopathological staining and analysis (Figure S19, Supporting Information). No significant change was found in the hematological and biochemical parameters, and no apparent pathological alterations were detected in major organs. After intravenous (i.v.) administration for 24 h, the biodistribution of ZIF‐8@MnO_2_ in the 4T1 tumor model was assessed (Figure S20, Supporting Information). The nanoparticles exhibited predominant distribution in the liver and kidney, with tumor accumulation reaching ≈3.5%ID g^−1^, potentially because of the enhanced permeability and retention (EPR) effect.^[^ ^23^ ^]^ These results indicated favorable biocompatibility and biosafety of ZIF‐8@MnO_2_ nanoparticles.

**Figure 6:**
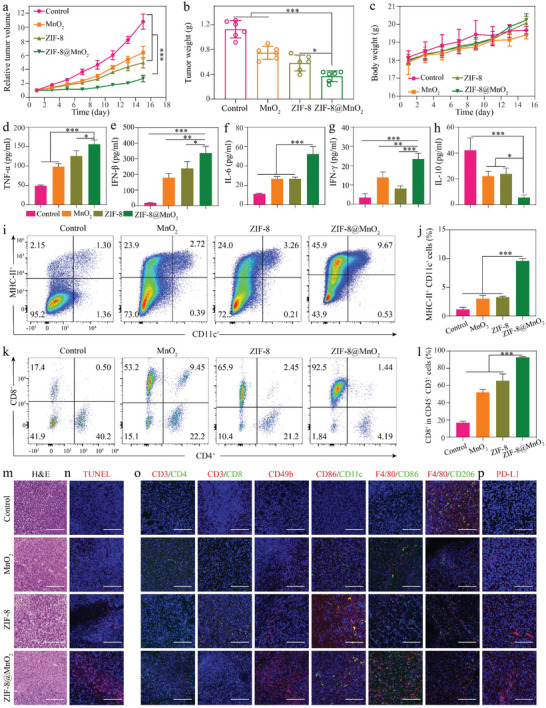
Therapeutic efficiency of ZIF‐8@MnO_2_ nanoparticles in vivo. a) Tumor growth curves after different treatments (*n* = 6, mean ± SD). b) The weight of 4T1(p53R249S) allograft tumors after different treatments (*n* = 6, mean ± SD). c) Body weight curves after different treatments (*n* = 6, mean ± SD). d–h) The cytokines of TNF‐α, IFN‐β, IL‐6, IFN‐γ, and IL‐10 in tumor tissue were detected by ELISA (*n* = 3, mean ± SD). i) Representative FACS data of the frequency of DC cells from mice in the differently treated group by flow cytometry. j) Quantified analysis of tumor‐infiltrating DC cells. k) Flow cytometric analysis of CD4^+^ and CD8^+^ T cells. l) Quantified analysis of CD8^+^ T cells. m) H&E; n) TUNEL immuno‐fluorescence staining after different treatments. Scale bar: 100 µm. o) CD4^+^ T cells, CD8^+^ T cells, DC cells, NK cells, M1 and M2 macrophages, and p) PD‐L1 immuno‐fluorescence staining after different treatments. Scale bar: 100 µm. One‐way ANOVA (b,d–h,j,l) and two‐way ANOVA analyses (a,c) were performed: *p < 0.05, **p < 0.01, ***p < 0.001.

Encouraged by the potent anti‐tumor activity demonstrated in vitro, in vivo therapy test of ZIF‐8@MnO_2_ was conducted by using 4T1 (p53R249S) allograft mice. Tumor‐bearing mice were randomly divided into four groups: PBS, MnO_2_, ZIF‐8, and ZIF‐8@MnO_2_. During the test period, no significant difference in body weight was observed among the four groups. However, the tumor growth in the ZIF‐8@MnO_2_ group was significantly restrained pertaining to volume and weight in comparison to the MnO_2_ group and ZIF‐8 group (**Figure** **6**a–c, Figure S21, Supporting Information). Furthermore, hematoxylin‐eosin (H&E) (Figure 6m) and TUNEL staining (Figure 6n) revealed evident nuclear condensation, fragmentation, and apoptosis‐related positivity of tumor cells after treatment with the nanoparticles.

To assess the immune activity in vivo, the production of immune‐related cytokines was measured using an ELISA assay. The levels of proinflammatory cytokines including TNF‐α, IFN‐β, IL‐6, and IFN‐γ were significantly increased after ZIF‐8@MnO_2_ treatment, while IL‐10 content was reduced (Figure 6d–h). The flow cytometry analysis revealed an increased number of CD8^+^ T cells and enhanced infiltration of DC cells after ZIF‐8@MnO_2_ treatment (Figure 6i–l; Figure S22, Supporting Information). The immuno‐fluorescence examination of tumor tissues also showed increased numbers of CD4^+^ and CD8^+^ T cells, higher activation levels of DC and NK cells, and remarkably skewed M2‐to‐M1 polarization of macrophages in the ZIF‐8@MnO_2_ group (Figure 6o). It is worth to mentioning that the expression of PD‐L1 was upregulated in the ZIF‐8@MnO_2_ group, indicating combinatory anti‐PD‐L1 immunotherapy (Figure 6p; Figure S23, Supporting Information). Taken together, these results suggested that ZIF‐8@MnO_2_ could partially reverse tumor immunosuppressive microenvironment and enhance the systemic anti‐tumor immune.

### 2.7 The Combination Effect of PD‐L1 Blocking Antibody With ZIF‐8@MnO_2_

**Figure 7:**
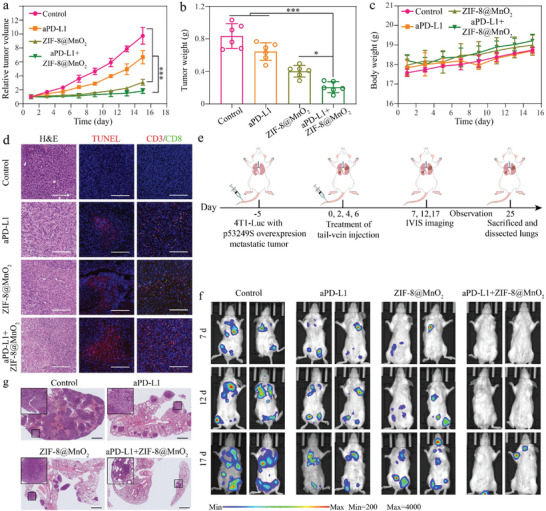
The combination effect of anti‐PD‐L1 blocking antibody with ZIF‐8@MnO_2_. a) Tumor growth curves after different treatments (PBS, aPD‐L1, ZIF‐8@MnO_2_, and aPD‐L1+ZIF‐8@MnO_2_) (*n* = 6, mean ± SD). b) The weight of 4T1 (p53R249S) allograft tumors after different treatments (*n* = 6, mean ± SD). c) Body weight curves after different treatments (*n* = 6, mean ± SD). d) H&E, TUNEL, and CD8^+^ T cells immuno‐fluorescence staining after different treatments. Scale bar: 100 µm. e) Schematic diagram of therapy: after 5 days intravenous injection of 4T1‐Luc (p53R249S) cells, four separate treatments were administered to BALB/c mice (*n* = 5), and f) IVIS Spectrum CT imaging was performed on days 7,12, and 17 post‐treatments, followed by g) H&E staining of lung tissues in sections. Scale bar: 100 µm. One‐way ANOVA (b) and two‐way ANOVA analyses (a,c) were performed: ***p < 0.001.

The upregulated PD‐L1 expression observed in the ZIF‐8@MnO_2_ group may be related to the activation of the cGAS‐STING pathway, resulting in the production of IFNs and downstream signaling molecules (such as IRF3) as the PD‐L1 transcription factors.^[^ ^24^ ^]^ To counteract the inhibitory effects of PD‐L1 expression on anti‐tumor immunity, the effect of PD‐L1 inhibitors in combination with ZIF‐8@MnO_2_ was worthy to be explored. Thus, we investigated the synergetic effect of anti‐PD‐L1 antibody (aPD‐L1) with ZIF‐8@MnO_2_ in vivo using a 4T1 (p53R249S) allograft model. Tumor‐bearing mice were randomly divided into four groups: PBS, aPD‐L1, ZIF‐8@MnO_2_, and aPD‐L1+ZIF‐8@MnO_2_. After 15 days of intravenous administration of different treatments, the tumor size and weight in aPD‐L1+ZIF‐8@MnO_2_ group were significantly reduced (**Figure** **7**a–c; Figure S24, Supporting Information). As shown by H&E and TUNEL staining, compared with the PD‐L1 inhibitor monotherapy, the combination therapy significantly improved tumor outcomes. The immuno‐fluorescence staining showed that the aPD‐L1+ZIF‐8@MnO_2_ group had the highest number of CD8^+^ T cells than the other groups, indicating a synergizing effect between ZIF‐8@MnO_2_ and the PD‐L1 inhibitor in the CD8^+^ T cell‐dominated anti‐tumor immune response (Figure 7d; Figure S25, Supporting Information). Last, the systematic immune effects of combining ZIF‐8@MnO_2_ and PD‐L1 inhibitors were investigated. The triple‐negative breast cancer (TNBC) is prone to systemic metastasis, especially lung metastasis.^[^ ^25^ ^]^ Therefore, breast cancer lung metastasis mice were established to evaluate the effect of combined aPD‐L1 treatment, which was induced by injecting 4T1‐Luc (p53R249S) cells into the tail vein. Imaging was acquired using IVIS Spectrum CT on days 7, 12, and 17 post‐treatments (Figure 7e). Compared to the other groups, the aPD‐L1+ZIF‐8@MnO_2_ group distinctly suppressed pulmonary metastasis (Figure 7f; Figure S26, Supporting Information), which was further confirmed by H&E staining of finding a prominent reduction in metastatic foci in the lungs on the 25th day after treatment (Figure 7g).

## 3 Conclusion

In summary, this study provided a nanotechnology‐based Mn–Zn dual‐ion delivery strategy to enhance the cGAS‐STING‐mediated anti‐tumor immunotherapy via. tumor‐acidic microenvironment and GSH responsive, cGAS enzymatic activity‐modulated, and mutp53‐targeted manifold pathways. Mechanistically, the synthesized ZIF‐8@MnO_2_ releases Zn^2+^ and Mn^2+^ in tumor acidic microenvironment with overexpressed GSH. The released Mn^2+^ can directly enhance cGAS sensitivity to dsDNAs and enzymatic activity. Meantime, the ROS generated by Zn^2+^ and mitochondrial damaged by Mn^2+^ provided more dsDNAs for activation of cGAS‐STING pathway. Notably, released Zn^2+^ could also induce the degradation of multifarious p53 mutants and decrease their inhibitory effects on the cGAS‐STING pathway. Both in vitro and in vivo results demonstrated that ZIF‐8@MnO_2_ effectively activated the cGAS‐STING pathway, enhanced the recruitment and activation of immune cells, especially in those tumors with mutated p53. The combination of PD‐L1 checkpoint inhibitors can further boost the immunotherapeutic effect, leading to the suppression of local tumors and distant metastases of breast cancer. We believe the proposed dual‐ion delivery strategy could serve as an approach for cGAS‐STING mediated tumor immunotherapy, especially rescuing mutp53‐driven tumors and expanding the applications of ion intervention therapy. More research about the impact of ZIF‐8‐based nanomaterials on the conformation of mutp53 should be carried out in the future.

## Conflict of Interest

The authors declare no conflict of interest.

## Supporting information

Supporting Information

## Data Availability

The data that support the findings of this study are available from the corresponding author upon reasonable request.
