# Supplementary material for: Nanoscale Metal–Organic Frameworks‐Mediated Degradation of Mutant p53 Proteins and Activation of cGAS‐STING Pathway for Enhanced Cancer Immunotherapy
Source: Adv Sci (Weinh). 2024 Jan 15;11(12):2307278. doi: 10.1002/advs.202307278 (PMC10966556; doi:10.1002/advs.202307278)
Supplement: Supplementary file 1 — Supporting Information [file ADVS-11-2307278-s001.pdf]

ADVANCED SCIENCE Open Access

Supporting Information

for *Adv. Sci.*, DOI 10.1002/advs.202307278

Nanoscale Metal–Organic Frameworks-Mediated Degradation of Mutant p53 Proteins and Activation of cGAS-STING Pathway for Enhanced Cancer Immunotherapy

Li Sun, Hongbo Gao, Han Wang, Jingwei Zhou, Xiuru Ji, Yuxin Jiao, Xiaojia Qin, Dalong Ni* and Xiangpeng Zheng**

Supporting Information

Nanoscale Metal-Organic Frameworks-Mediated Degradation of Mutant p53 Proteins and Activation of cGAS-STING Pathway for Enhanced Cancer Immunotherapy

Li Sun[#], Hongbo Gao[#], Han Wang, Jingwei Zhou, Xiuru Ji, Yuxin Jiao, Xiaojia Qin, Dalong Ni* and Xiangpeng Zheng**

Experimental Section

Materials: Zinc acetate and 2-methylimidazole (98%) were provided by Macklin Inc. (Shanghai China). Potassium permanganate ($KMnO_4$), ethanol, and 30% v/v hydrogen peroxide were purchased from Sinopharm Chemical Reagent Co., Ltd (Beijing, China). GSH and GSSG Assay Kit, 4',6-diamidino-2-phenylindole (DAPI), Mito-Tracker Deep Red FM, hematoxylin-eosin reactive, oxygen species assay Kit, JC-1 detection kit, Calcein-AM/PI detection kit, Annexin V-FITC/PI detection kit and TUNEL apoptosis assay kit were supplied by Biotechnology Co., Ltd. (Shanghai, China). Matrigel was purchased from BD Biosciences （MA, USA), Transwell assays were performed by using Transwell plates (Corning, USA). Probenecid and 4% paraformaldehyde were provided by Sigma (CA, USA). Metal Fluor™ Zn-520 was purchased from AAT Bioquest Inc. (Sunnyvale, USA). Cell Counting Kit 8 (CCK8) was obtained from Abcam (MA, USA). Antibodies used in these studies were obtained as follows: Anti-p53 (DO-1) was purchased from Santa Cruz Biotechnology (TX, USA). The dsDNA monoclonal antibody was provided by Cayman (MI, USA). FITC-labeled goat anti-mouse IgG H&L antibody, anti-CD11b antibody, anti-CD163 antibody, anti-CD3 antibody, anti-CD3 epsilon antibody, anti-CD4 antibody, anti-CD8 alpha antibody, and anti-integrin alpha 2 antibody were obtained from Abcam (MA, USA). Human and mouse reactive STING pathway antibody sampler Kit, β-Actin rabbit mAb, GAPDH rabbit mAb, anti-mouse IgG, HRP-linked antibody, cleaved caspase-9 rabbit mAb, Bax rabbit mAb, ubiquitin (P4D1) mouse mAb, k48-linkage specific polyubiquitin rabbit mAb, CD11c rabbit mAb, PD-L1 rabbit mAb, anti-rabbit IgG, HRP-linked antibody, and mouse anti-rabbit IgG (conformation specific) mAb (HRP Conjugate) were purchased from Cell Signaling Technology (CST, Carlsbad, USA).

Pierce® classic magnetic IP/Co-IP kit was provided by Thermo Fisher. BV605 rat anti-mouse I-A/I-E, BV421 hamster anti-mouse CD11c, BB700 rat anti-mouse CD8a, PE rat anti-mouse CD4, BV510 rat anti-mouse CD45, BV786 rat anti-mouse CD86, Alexa Fluor® 700 hamster anti-mouse CD3e were obtained from BD bioscience (San Jose, CA).

Characterization: Transmission electron microscopy (TEM) was conducted by FEI Talos F200X (FEI, Hillsboro, OR, USA). X-ray diffraction (XRD) analyses were performed using a Rigaku D/MAX-2250V diffractometer (Rigaku Instrument, Tokyo, Japan). The hydrodynamic diameter and zeta potential were determined through dynamic light scattering (DLS, Malvern, UK). Fourier transform infrared (FT-IR) spectroscopy was carried out with Thermo Fisher Nicolet 6700 (Waltham, MA, USA). The binding energy was evaluated using an ESCALAB 250Xi X-ray photoelectron spectrometer (Thermo Fisher Scientific, USA). Ultraviolet-visible (UV-Vis) spectrum was measured by Shimadzu UV-3600 Plus. The content of Mn and Zn was measured by inductively coupled plasma optical emission spectrometry (ICP-OES, Agilent 725). The content of Zn was determined by inductively coupled plasma mass spectrometry (ICP-MS) (Perkin Elmer, USA). The cell viability was assessed using the CCK8 assay and measured with a BioTek microplate reader (WI, USA) at a wavelength of 450 nm. Flow cytometry analysis was conducted using a BD FACS Calibur Flow Cytometer (MA, USA) equipped with FACS Diva software. Confocal laser scanning microscopy (CLSM) images were acquired with an SP8 LEICA microscope. The *in vivo* imaging of animals was performed using the IVIS Spectrum CT (NexION 2000, Perkin Elmer, MA, USA).

Preparation of ZIF-8: The synthesis of ZIF-8 nanoparticles was conducted according to the modified method described previously.[1] Firstly, zinc acetate (1 mmol) was dissolved in 2 mL of deionized water (ddH$_2$O). Simultaneously, 2-methylimidazole (50 mmol) was dissolved separately in 20 mL of ddH$_2$O. Subsequently, the solution of zinc acetate was slowly added to the 2-methylimidazole solution under vigorous stirring, and the mixture was allowed to react for 30 min. The resulting product was collected by centrifugation and washed with ethanol. Finally, the ZIF-8 nanoparticles were suspended in ddH$_2$O for further applications.

Preparation of MnO$_2$: MnO$_2$ nanoparticles were synthesized by directly mixing KMnO$_4$ and BSA solution. Firstly, 50 mg of BSA powder was dissolved into 9 mL of ddH$_2$O to form a clarified solution. Next, 1 mL of KMnO$_4$ at a concentration of 10 mM was added dropwise to the above BSA solution under stirring. The reaction was continued for 30 min at room temperature (RT) to obtain a brownish-yellow solution. The solution was dialyzed with a dialysis bag for 48 h to remove unreacted molecules and stored at 4 °C for subsequent characterization and use.

Preparation of ZIF-8@MnO_2: A solution of $KMnO_4$ (1 mg/mL, 4 mL) was added to the aqueous solution containing ZIF-8 nanoparticles (5 mg/mL, 10mL), followed by vigorous stirring at RT. After a duration of 30 min, the resulting hybrid nanoparticles, comprising a ZIF-8@MnO_2 core-satellite structure, were washed with ddH_2O and subsequently re-dispersed in water.

Zinc and Mn ions release: ZIF-8@MnO_2 (Zn, 841 ppm) was dispersed in 400 mL of PBS buffer (pH = 7.4, GSH 0 mM or pH = 6.0, GSH 10 mM) within a dialysis bag and subsequently incubated for various durations. Then, the suspension was collected and diluted with ultrapure water before the ICP-OES analysis.

Cell culture: The human breast cancer cell line MCF7 (WTp53), MDA-MB231 (p53R280K), and mouse breast cancer cell line 4T1 (p53 null), 4T1 (p53R249S) were cultured in RPMI-1640 medium with 10% FBS. The human breast cancer cell line BT549 was cultured in RPMI-1640 medium with 10% FBS and 0.023 IU/ml insulin. The SKBR3 human breast cancer cell line was cultured in McCoy's 5A Medium Modified with 10% FBS. All cell lines were obtained from the American Type Culture Collection (ATCC) and cultured under 5% CO_2 at 37 °C.

SC-ICP-MS analysis: The content of Zn in cells was detected using a Perkin Elmer NexION 2000 ICP-MS. Briefly, cells were treated with $ZnCl_2$ or ZIF-8@MnO_2 for 4 h, followed by washing with PBS three times and fixation with paraformaldehyde overnight, before being subjected to detection.

Construction of p53-mutant cell line: p53R249S overexpression lentivirus was constructed using Ubi-MCS-3FLAG-SV40-EGFP-IRES-Hygromycin vector (Jikai Genechem Co., Ltd, Shanghai, China). The lentivirus was transfected to 4T1 cells according to the manufacturer's instructions and screened with 250 μg/mL hygromycin.

Cell viability: Cytotoxicity of ZIF-8@MnO_2 at different concentrations on BT549, MB231, SKBR3, and MCF7 cell lines was evaluated using CCK8 assay, and the biosafety of nanoparticles was assessed on three normal tissue cell lines: Human embryonic renal cell line 293T, rat cardiomyocytes H9C2 and mouse hippocampal neuronal cells HT22. Cells (5 × 10^3 per well) were dispersed in 96-well plates and different concentrations of nanoparticles solution were added, CCK8 reagent was added according to the instructions after 24 h of treatment, and the absorbance values were measured at 450 nm.

GSH-activated MRI *in vitro* and *in vivo*: To validate the performance of the synthesized nanomaterials for T_1-weighted MRI, different concentrations of ZIF-8@MnO_2 (Mn: 0.0125, 0.0250, 0.0500, 0.1000, and 0.2000 mM) were utilized to assess its relaxation rate at pH 7.4 or pH 6.0 (containing 1 mM GSH). For *in vivo* imaging, ZIF-8@MnO_2 (Mn: 5 mg/kg) was injected

into 4T1 tumor-bearing mice via tail vein, followed by MRI scans (3-T Trio scanner, Siemens) at different time intervals.

Detection of intracellular Zn^{2+}: Metal Fluor™ Zn-520 as cell-permeant AM-ester for detecting intracellular Zn^{2+} level has shown great fluorescence enhancement upon binding Zn^{2+}. After incubation with Zn^{2+} or ZIF-8@MnO_2 (Zn, 40ppm) for 8 h, BT549 cells were stained with Zn^{2+} dyes (5 μM) and DAPI serially. Subsequently, cells were treated with 1.0 mM probenecid. Fluorescence images were acquired using CLSM at Ex/Em = 492/514 nm.

Detection of intracellular GSH and GSSG: The GSH/GSSG ratio was measured using the GSH and GSSG assay kit. Cells were collected by centrifugation after washing in PBS, and a solution of protein removal reagent M equal to three times the volume of cell sedimentation was added for thorough vortex mixing. Next, the samples were subjected to two rapid freeze-thaw treatments using liquid nitrogen and 37 °C water bath, and then placed in 4 °C or ice bath for 5 min. The samples were centrifuged at 4 °C and 10,000 g for 10 min and the supernatant was taken as a sample for total GSH determination. A portion of the prepared sample to be measured for total GSH content was added proportionally to a mixture of GSH scavenging aid and GSH scavenging reagent working solution, followed by immediate vortex mixing, and the reaction was carried out at 25 °C for 60 min. Then incubation with GSH working solution for 5 min. After the NADPH solution was added, the absorbance value (A412) was measured immediately using an enzyme meter.

Detection of intracellular ROS: BT549 cells were seeded in confocal dishes at a density of 4 × 10^5 cells per dish and incubated for 24 h. All cells were incubated with PBS, MnO_2, ZIF-8, and ZIF-8@MnO_2 (Zn, 40 ppm) for 8 h. After removing the culture medium, the cells were washed three times with PBS. Subsequently, the cells were treated with the 5 μM DCFH-DA solution and incubated at 37 °C for 30 min protected from light. Fluorescence images were obtained using CLSM with an Ex/Em wavelength of 504/529 nm.

Detection of mitochondrial membrane potential: JC-1 undergoes aggregation within the mitochondrial matrix, forming polymers known as J-aggregates with high mitochondrial membrane potential, which emit red fluorescence. Conversely, JC-1 remains in a monomeric state and emits green fluorescence with low mitochondrial membrane potential. All cells were incubated with PBS, MnO_2, ZIF-8, and ZIF-8@MnO_2 (Zn, 40 ppm) for 8 h. Following the addition of JC-1 staining working solution, cells were incubated at 37 °C for 20 min. Subsequently, the BT549 cells were washed with JC-1 staining buffer and immediately assayed. The fluorescence signals of monomers were detected at Ex/Em = 514/529 nm, while the fluorescence signals of J-aggregates were detected at Ex/Em = 585/590 nm.

Detection of cytoplasmic dsDNA: BT549 cells were seeded in confocal dishes overnight. All cells were incubated with PBS, MnO_2, ZIF-8, and ZIF-8@MnO_2 (Zn, 40 ppm) for 8 h. The cell culture was replaced with Mito-Tracker Deep Red FM working solution and incubated at 37 °C for 20 min. Subsequently, the stained cells were fixed and permeabilized, which were incubated overnight with dsDNA monoclonal antibody. Then, FITC-labeled goat anti-mouse IgG H&L secondary antibody was performed for 1 h and the fluorescence images were obtained using CLSM.

Immuno-fluorescence assays: The effect of nanoparticles on p53 protein in breast cancer cell lines with different mutation status was verified using p53 (DO-1) immuno-fluorescence. BT549, MB231, SKBR3, and MCF7 cells were seeded in confocal dishes for 12 h. All cells were incubated with PBS, MnO_2, ZIF-8, and ZIF-8@MnO_2 (Zn, 40ppm) for 8 h, which were then fixed in 4% paraformaldehyde for 10 min at RT. Next, 0.25% Triton-X 100 was used to increase cell permeability. After blocking with 5% bovine serum albumin (BSA), cells were incubated with a primary antibody p53 antibody (DO-1) overnight at 4 °C, and incubated with FITC-labeled goat anti-mouse IgG H&L secondary antibody for another 1 h. Finally, cells were stained with DAPI for 5 min to visualize nuclei. Confocal microscopy images were collected using CLSM.

Cell migration and invasion assay: BT549 cells were inoculated in 6-well plates and cultured overnight. After adhering and growing to confluence, all cells were incubated with PBS, MnO_2, ZIF-8, and ZIF-8@MnO_2 (Zn, 40 ppm) for 8 h at 37 °C. Next, vertical scratches on the cell layer were made using a 200 μL pipette tip. Then, cells were washed with PBS to eliminate moving cells. Scratched pictures were captured at 0 h, 24 h, and 48 h. Cell invasiveness was determined by using the matrigel-coated Transwell. BT549 cells (1.0×10^5 cells per well) were placed in the upper chamber without FBS while RPMI-1640 medium containing 10% FBS was added to the lower chamber. After incubation at 37 °C for 24 h and 48 h, cells on the lower surface of the membrane were fixed using 4% paraformaldehyde and stained with 0.1% crystal violet, and randomly selected fields of view were used to count the cells under an inverted microscope.

Cell apoptosis: BT549 cells in the logarithmic growth phase at a concentration of 1×10^6 cells/mL were plated in a 6-well plate. Upon adherence, the cells were treated with PBS, MnO_2, ZIF-8, and ZIF-8@MnO_2 (Zn, 40 ppm) for 8 h. Following the culture medium change, cells were further incubated for 12 h. The medium and cells were then collected and centrifuged at 800 rpm for 5 min. Subsequently, cells were resuspended in a binding solution containing 5 μL

of Annexin V-FITC and 10 μL of PI, and incubated at 37 °C for 15 min. Cell apoptosis rate was promptly analyzed using a flow cytometer (Beckman Coulter, USA).

RNA extraction and RT-qPCR: Total RNA from cells/tissues extraction, and RT-qPCR were performed as previously described.[2] Briefly, RNA was isolated using Trizol reagent (Invitrogen) and RNA purification kit (EZBioscience) protocols with some modifications. After the quantity and quality of RNA were confirmed with a NanoDrop spectrophotometer, cDNA was synthesized using a color reverse transcription Kit with gDNA remover (EZBioscience). RT-qPCR was performed on a light cycler 480 instruments (Roche) using 2 × Color SYBR Green qPCR Master Mix (EZBioscience). GAPDH was used as an endogenous control.

Immunoblotting: The protein expression levels of the cGAS-STING pathway, p53, cleaved caspase-3, cleaved caspase-9, cytochrome c (Cyto-C), GAPDH, β-Actin, and BAX were detected by immunoblotting. Briefly, cells or tumor-bearing mice were treated with PBS, MnO_2, ZIF-8, and ZIF-8@MnO_2 or inhibitors and agonists, respectively. Tumor cells were collected and lysed with lysate. After determining the total protein concentration, cellular proteins were separated by sodium dodecyl sulfate-polyacrylamide gel electrophoresis (SDS-PAGE) and transferred to a polyvinylidene fluoride membrane (PVDF). After blocking in 5% BSA, the membranes were incubated with specific primary antibodies overnight at 4 °C, which were further incubated with HRP-goat anti-rabbit immunoglobulin G (IgG) for another 1 h. Finally, protein detection was performed using an enhanced ECL luminescence solution reaction (Thermo Fisher Scientific, USA). The above method refers to the previous description.[3]

Coimmunoprecipitation (CoIP): For the CoIP assay, cells were washed with pre-cooled PBS, harvested, and lysed in immunoprecipitation buffer (pH 7.4, 0.025 M Tris, 0.15 M NaCl, 0.001 M EDTA, 1% NP40, 5% glycerol) for 5 min. Lysates were clarified by centrifugation at 13,000 g (4 °C) for 20 min and incubated with the antibodies overnight at 4 °C to form an immune complex, followed by incubation with protein A/G beads for 1 h at RT to form an antigen/antibody complex. Subsequent immunoblotting was performed after protein elution and denaturation according to the previous method.[4]

Biosafety evaluation *in vivo*: All animal experiments were conducted following the guidelines of the Animal Experimental Ethics Committee and approved by the Animal Experimental Ethics Committee of Fudan University-affiliated Huadong Hospital (approval number: 202209017S). Healthy BALB/c mice (18-22g, 5-6 weeks old, female, SPF grade) were purchased from Gempharmatech Co., Ltd. The BALB/c mice were randomly divided into four groups (n = 3) and then intravenously injected with PBS, MnO_2, ZIF-8, and ZIF-8@MnO_2 (Zn, 6mg/kg). On the 15th day post-injection, blood samples were collected for hematological and

biochemical analysis, and major organ tissues were histopathologically analyzed using H&E staining.

Tumor model establishment and anticancer therapy: The tumor model was established by dispersing 2 × 10^6 4T1(p53R249S) cells in PBS and implanted into the right side of the fourth pair of fat pads of BALB/c mice. When the volume reached 100-150 mm^3, tumor-bearing mice were randomly divided into 4 groups (*n* = 6) and injected with PBS, MnO_2, ZIF-8, and ZIF-8@MnO_2 (Zn, 6 mg/kg). Tumor volume and body weight were measured every other day. After 15 days of treatment, transplanted tumors were excised for further analysis. For the lung metastasis model, 5 × 10^5 4T1-Luc (p53R249S) cells in 100 μL PBS were injected into mice via the tail vein to generate lung metastases. Subsequently, mice were randomly divided into 4 groups (*n* = 5) and treated with PBS, aPD-L1 (2 mg/kg), ZIF-8@MnO_2 (Zn, 6mg/kg), and aPD-L1+ ZIF-8@MnO_2. IVIS Spectrum CT imaging was performed on days 7, 12, and 17 post-treatments, followed by staining of lung tissues in sections.

RNA-seq analysis: To further elucidate the gene expression levels and changes within tumor cells, the tumor-bearing mice were randomly divided into two groups (*n* = 5), and treated with PBS and ZIF-8@MnO_2 respectively. Subsequently, samples were collected for transcriptomic analysis according to the previous methods.[5] Firstly, RNA was extracted from the tissues. Then, the quality of the RNA sample was assessed and quantified before constructing the sequencing library. Gene set enrichment analysis (GSEA) was performed by comparing the tumor samples treated with PBS to evaluate the gene set enrichment. Firstly, genes were sorted, and the enrichment score (ES) was calculated. Then, the significance level of the ES was estimated, followed by multiple hypothesis testing. The criteria for statistical significance were defined as FDR < 0.25, $p < 0.05$, and |NES| > 1.

Cytokine ELISA assays: The cytokines of TNF-α, IL-6, IFN-γ, IFN-β, and IL-10 in tumor tissue were detected by ELISA. The samples underwent a PBS rinse to eliminate remaining blood or impurities on the tissue surface. Following the weighing of tissue blocks, pre-cooled PBS (with protease inhibitor added before use) was added in a certain ratio (tissue weight: PBS volume = 1:9). After homogenization on ice, centrifugation was performed at 5000 g for 10 min at 4 °C, the supernatant was extracted according to the manufacturer's instructions of the ELISA kit, and the results were analyzed using the ELISACalc Fitting Data Software.

Flow cytometry analysis on tumor tissues: Briefly, the tissue was dissected into small pieces and transformed into the cell suspension, followed by cell lysis and individual cell filtration by a screen. Subpopulations of cells were then stained with antibodies or fluorescent markers to distinguish cell types and specific cell surface markers. Finally, cell analysis was carried out by

injecting stained cell samples into a flow cytometer. Data processing was performed by the FlowJ software.

Tumor section staining: The tumor tissue was fixed with 4% paraformaldehyde and embedded in paraffin, and then the paraffin sections were stained with H&E and TdT-mediated dUTP nickel end labeling (TUNEL) kit. The infiltration of immune cells was detected by fluorescence staining. Tumor tissue was embedded in OCT in a cryomold and sectioned using a cryosectioner. Sections were air-dried and fixed in 4% paraformaldehyde for 10 min. After permeabilized and blocked, slices were incubated with immune-specific antibodies (CD8, CD3, CD4, CD49b, CD11c, CD86, CD206, F4/80, PD-L1) overnight, and further incubated with 594/647 nm fluorescent secondary antibodies for 1 h at RT and mounted with anti-fade DAPI. Images were captured using a Zeiss 780 upright confocal microscope.

Statistical Analysis: The measurement data were expressed as the mean ± standard deviation (SD). Statistical analysis was performed by two-tailed Student's t-test for comparison between two groups and one-way analysis of variance (ANOVA) followed by Turkey's post-test for comparison of three or more groups. $*p < 0.05$, $**p < 0.01$, $***p < 0.001$.

Primers used for RT-qPCR:

Gene name	Sequence (5'-3')
h-IFNB1-F	GTCAGAGTGGAAATCCTAAG
h-IFNB1-R	TATGCAGTACATTAGCCATC
h-ISG56-F	GCCTTGCTGAAGTGTGGAGGAA
h-ISG56-R	ATCCAGGCGATAGGCAGAGATC
h-GAPDH-F	CTCTGCTCCTCCTGTTCGAC
h-GAPDH-R	GCGCCCAATACGACCAAATC
m-GAPDH-F	TGATGGGTGTGAACCACGAG
m-GAPDH-R	TAGGGCCTCTCTTGCTCAGT
m-Ccl2-F	CACTCACCTGCTGCTACTCA
m-Ccl2-R	GACCTTAGGGCAGATGCAGT
m-Ccl7-F	CTTCTGTGCCTGCTGCTCATAG
m-Ccl7-R	CTGGTGATCCTTCTGTAGCTCTTG
m-Ccl6-F	CTGCTGCTTCTCTTATGCCACAC
m-Ccl6-R	CCCTCCTGCTGATAAAGATGATGC
m-Ccl9-F	CAGTCTGAAGGCACAGCAAGG
m-Ccl9-R	GTGAGTTATAGGACAGGCAGCAATC
m-Ccl11-F	GCACCCTGAAAGCCATAGTCTTC
m-Ccl11-R	CATCCTGGACCCACTTCTTCTTG
m-Ccl3-F	TTCTGCTGACAAGCTCACCC
m-Ccl3-R	GTCTCTTTGGAGTCAGCGCA
m-Il33-F	TGCTACTACGCTACTATGAGTCTCC
m-Il33-R	TCATGTTCACCATCAGCTTCTTCC
m-Il6-F	GTCTTCTGGAGTACCATAGC
m-Il6-R	TATCTGTTAGGAGAGCATTG
m-Ifit1-F	CTCTGAAAGTGGAGCCAGAAAAC
m-Ifit1-R	AAATCTTGGCGATAGGCTACGA
m-Ifit3-F	TTCCCAGCAGCACAGAAAC
m-Ifit3-R	AAATTCCAGGTGAAATGGCA
m-Ifit3b-F	TGCGATCCACAGTGAACAACAG
m-Ifit3b-R	TCGTCTCAGTTCTGCCATCCTC
m-Ifit2-F	CTGAAGCTTGACGCGGTACA
m-Ifit2-R	ACTTGGGTCTTTCTTTAAGGCTTCT
m-Ifit44l-F	AGATGGTGACCGCCGAACAG
m-Ifit444l-R	CGACTTTCCCTGAGCCTACTGG
m-Cxcl3-F	GAAGTCATAGCCACTCTCAAGGATG
m-Cxcl3-R	TTGCCGCTCTTCAGTATCTTCTTG
m-Cxcl12-F	AGAGCCACATCGCCAGAGC
m-Cxcl12-R	ATCCACTTTAATTTCGGGTCAATGC
m-Irf7-F	CAATTCAGGGGATCCAGTTG
m-Irf7-R	AGCATTGCTGAGGCTCACTT
m-Mx1-F	GTCTTGGATGTGATGCGGAACC
m-Mx1-R	TTGGATGTCCTGCTGACCTCTG
m-Oasl1-F	AGGTAGGCTGCTTTGGGAATGG
m-Oasl1-R	CCTGATGGTGCTTGGCTTCTTC
m-Oas2-F	CTGGAACTGCTCACTGTGTATGC
m-Oas2-R	ACGGTCCTGACGCCTTGTG

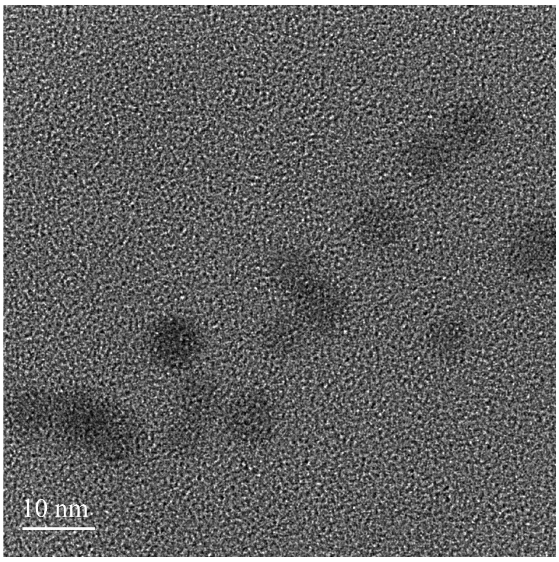

Figure S1. The TEM image of MnO_2 nanoparticles.

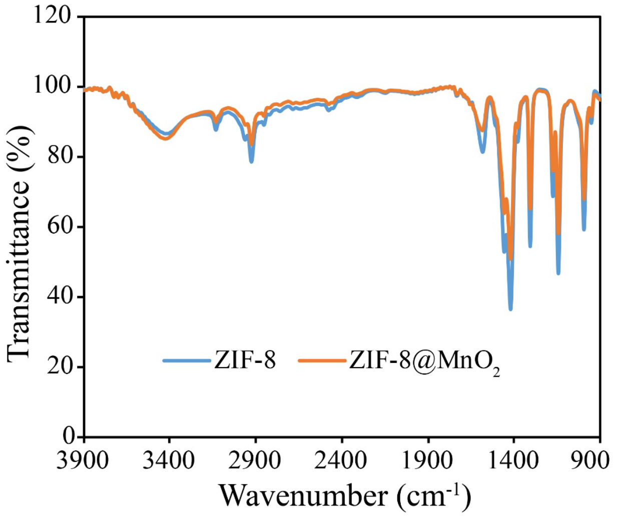

Figure S2. The FTIR spectra of ZIF-8 and ZIF-8@MnO_2 nanoparticles.

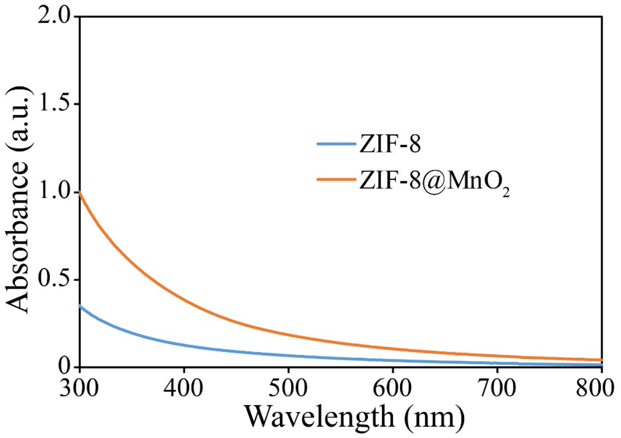

Figure S3. UV-vis of ZIF-8 and ZIF-8@MnO_2 nanoparticles.

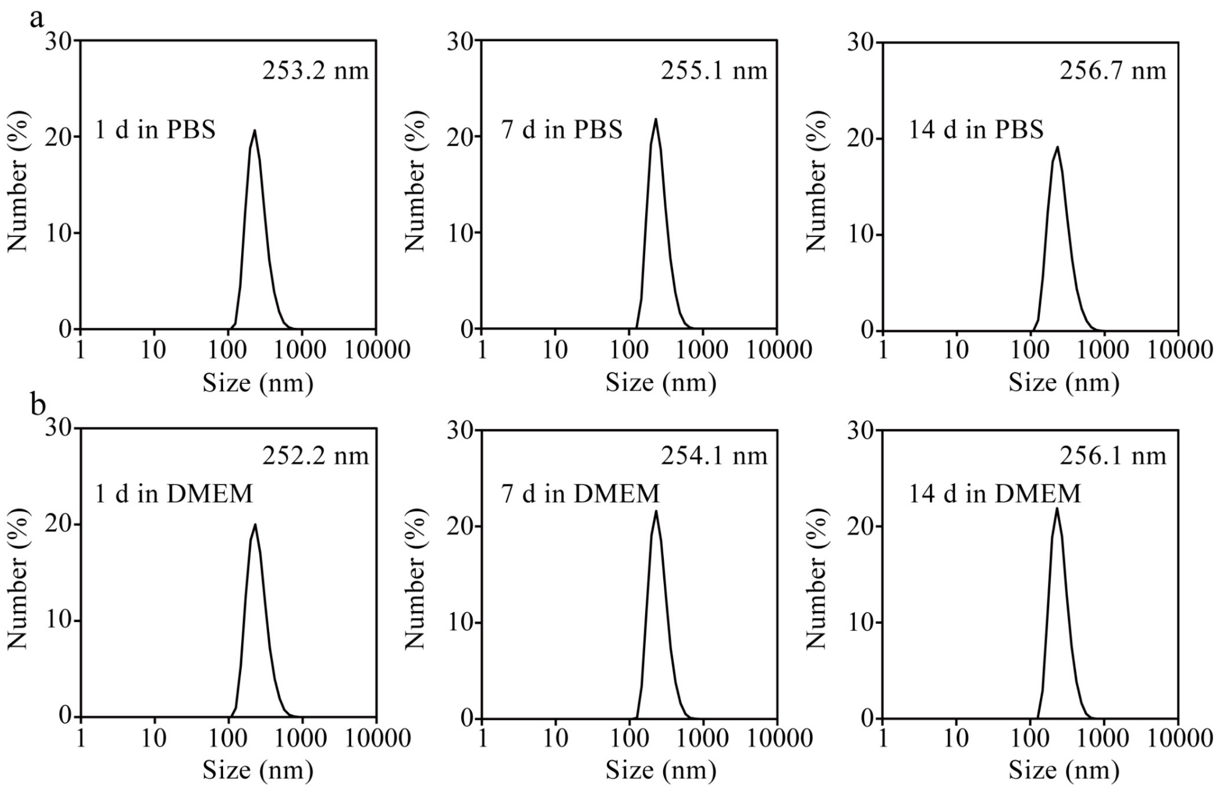

Figure S4. The stability of ZIF-8@MnO_2. The size change of ZIF-8@MnO_2 in a) PBS and b) DMEM on the 1, 7, and 14th day.

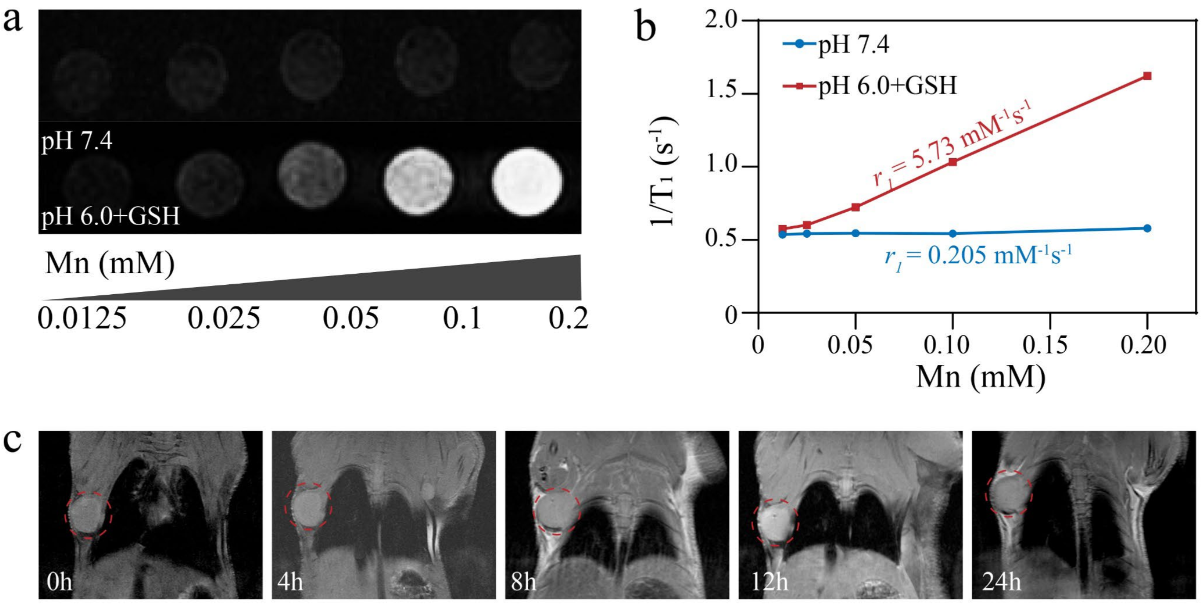

Figure S5. a) T_1-weighted MR images of ZIF-8@MnO_2 with different concentrations under PBS of pH 7.4 and pH 6.0 (containing 1 mM GSH). b) The r_1 value of ZIF-8@MnO_2 with or without GSH. c) T_1-weighted MR images of 4T1 tumor after injection with ZIF-8@MnO_2 at different time points.

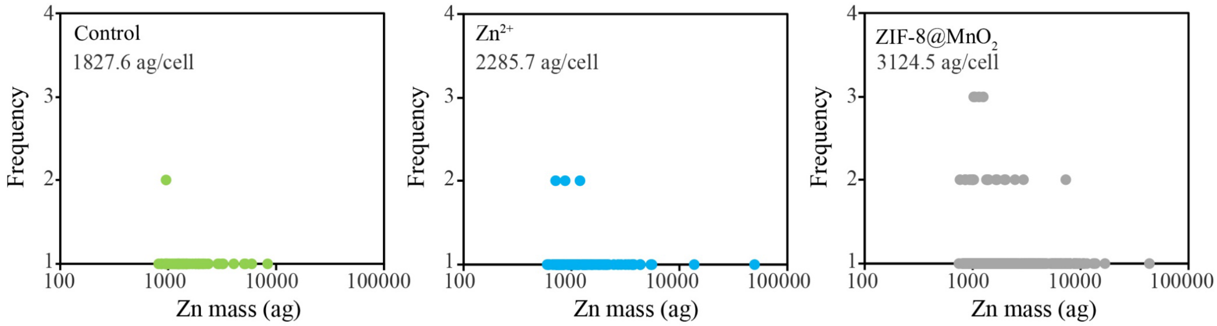

Figure S6. Zn content in BT549 cells after different treatments.

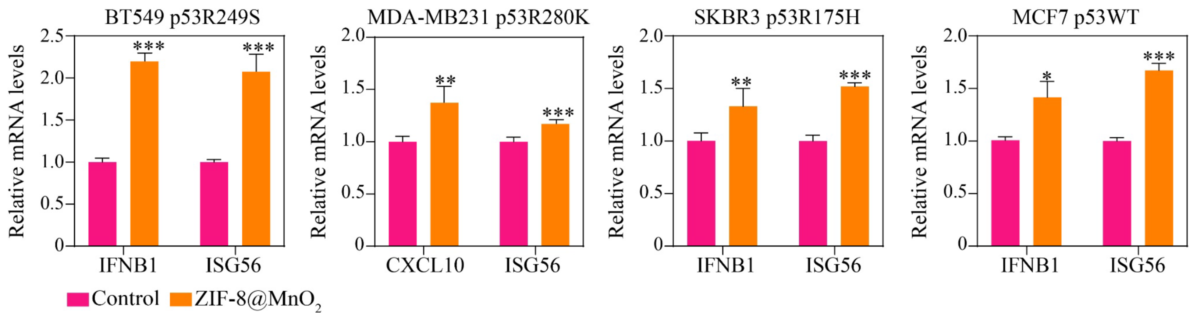

Figure S7. RT-qPCR analysis of target genes expression in cells (BT549, MB231, SKBR3, MCF7) treated with ZIF-8@MnO_2 (n = 3, mean ± SD). Statistical analysis was calculated by Student's t-test: *$p < 0.05$, **$p < 0.01$, ***$p < 0.001$.

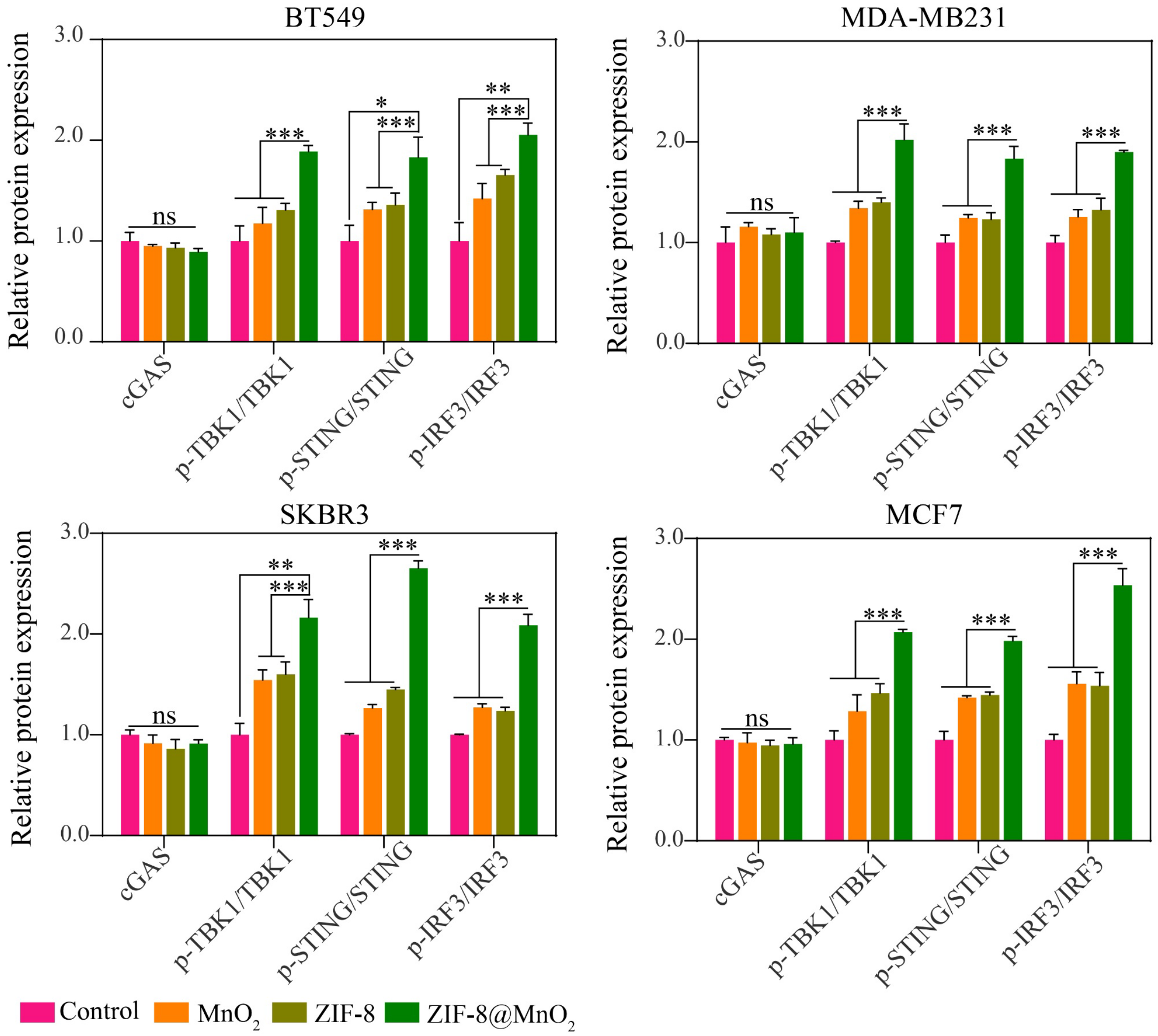

Figure S8. Grayscale analysis of the western blot bands of BT549, MDA-MB231, SKBR3, and MCF7 cells with different treatments ($n = 3$, mean ± SD). Statistical analysis was calculated by one-way ANOVA analyses: $^*p < 0.05$, $^{**}p < 0.01$, $^{***}p < 0.001$.

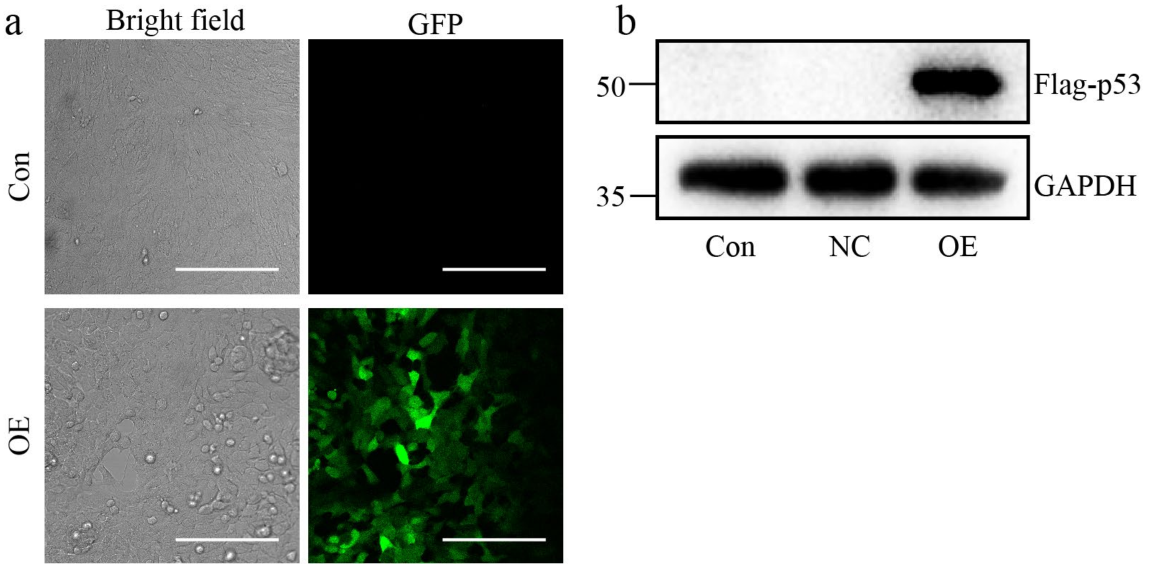

Figure S9. The construction of 4T1 (p53R249S) cells. a) Confocal microscopy images of 4T1 cells with or without transfected Flag-and GFP-tagged-p53R249S. Scale bar = 100 µm. b) Western blotting analysis of target protein overexpression.

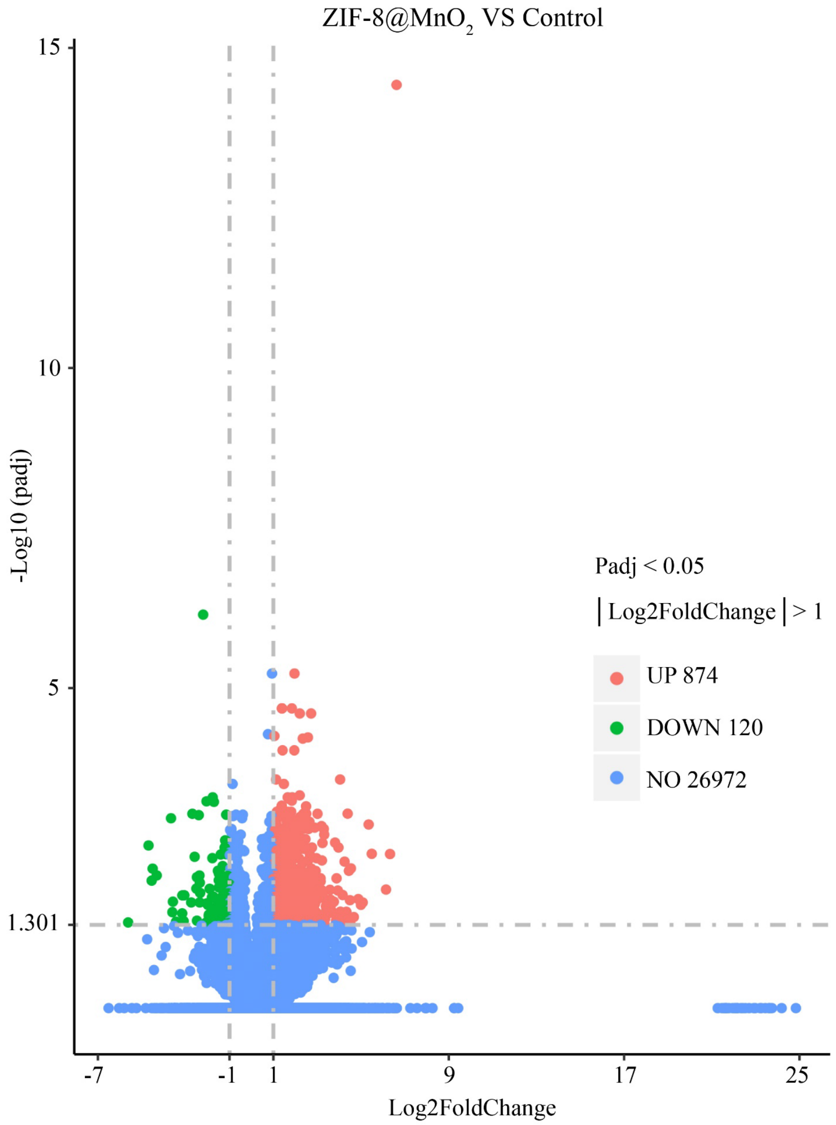

Figure S10. Volcano plot showing differentially expressed genes between sexes.

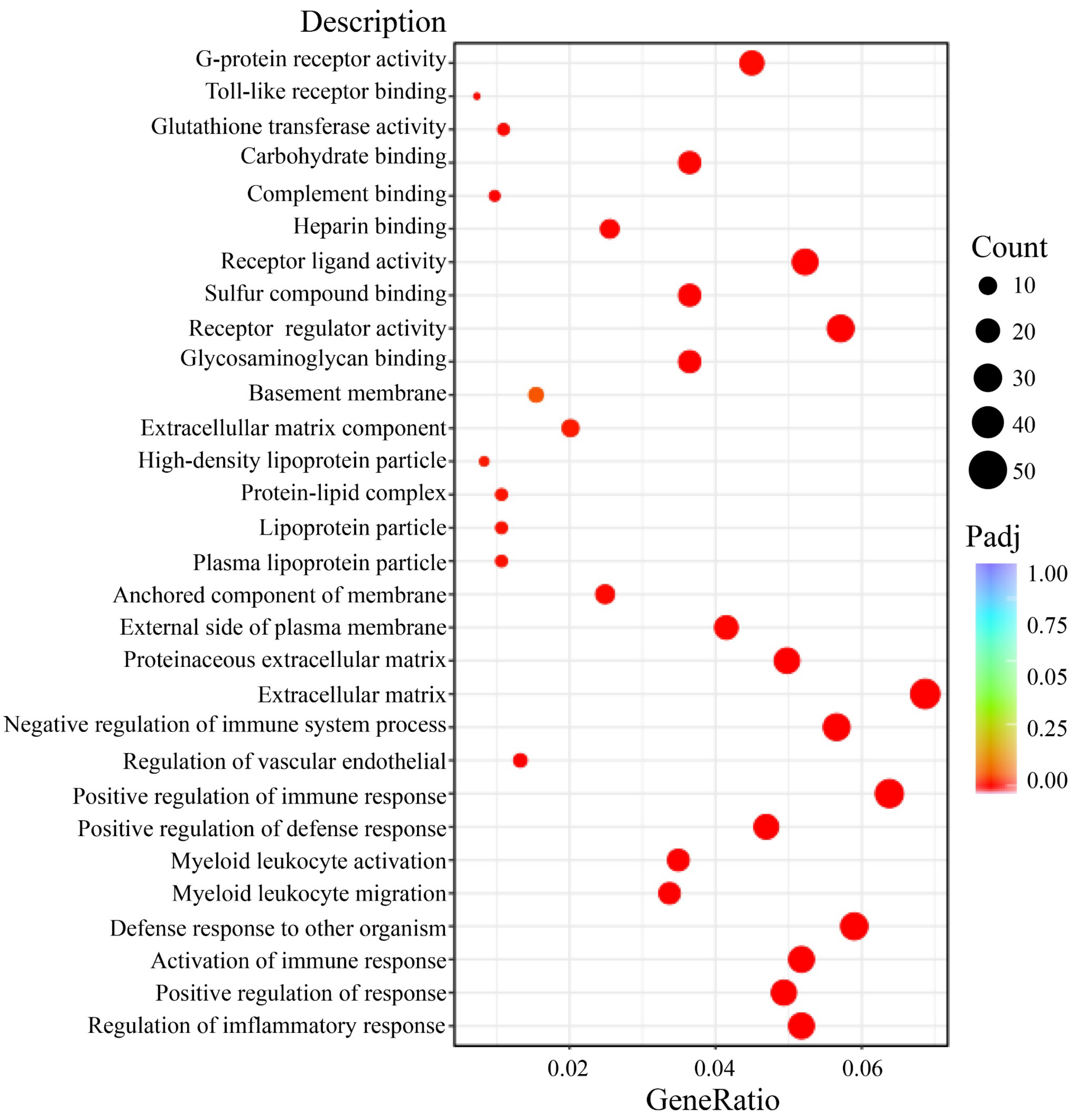

Figure S11. GO functional enrichment analysis of differentially expressed genes was used to identify GO functional items.

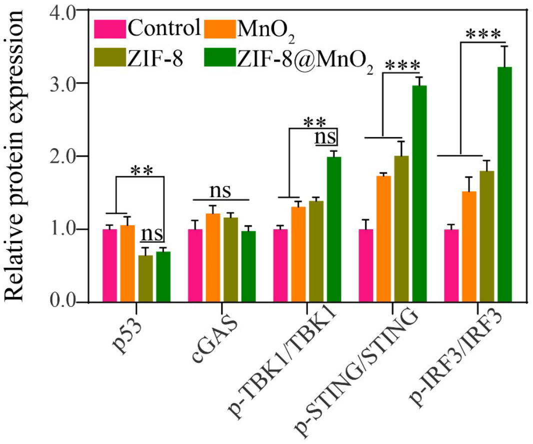

Figure S12. Grayscale analysis of the western blot bands of 4T1 (p53R249S) tumors with different treatments ($n = 3$, mean ± SD). Statistical analysis was calculated by one-way ANOVA analyses: $^{**}p < 0.01$, $^{***}p < 0.001$.

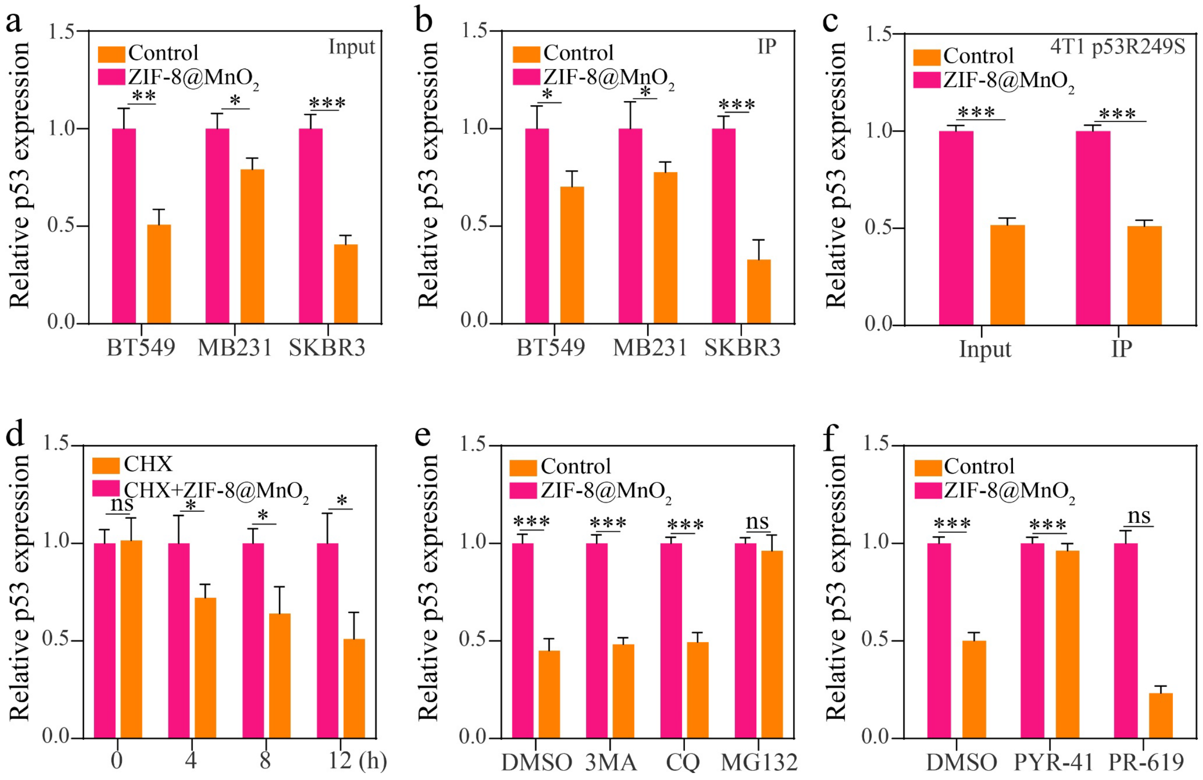

Figure S13. Grayscale analysis of the western blot bands of Coimmunoprecipitation (CoIP) with different treatments. The grayscale analysis of western blot bands of a) input and b) immunoprecipitation (IP). c) The grayscale analysis of western blot bands of input and IP of 4T1 (p53R249S). The grayscale analysis of western blot bands of BT549 cells with the treatment of d) CHX, e) 3MA, CQ, and MG132, and f) PYR-41 and PR-619 (n = 3, mean ± SD). Statistical analysis was calculated by Student's t-test: *$p < 0.05$, **$p < 0.01$, ***$p < 0.001$.

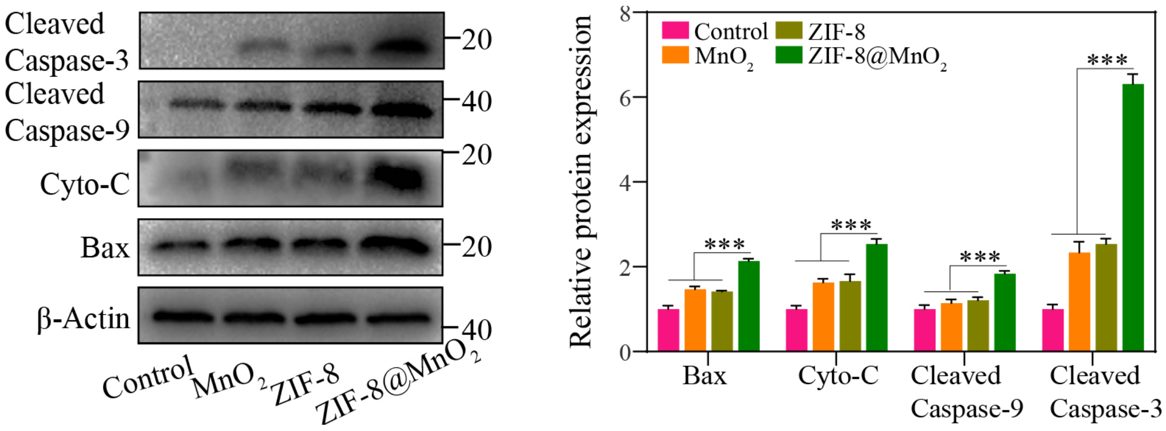

Figure S14. Western blotting to detect the expression of apoptosis-related proteins ($n = 3$, mean ± SD). One-way ANOVA analyses were performed: ***$p < 0.001$.

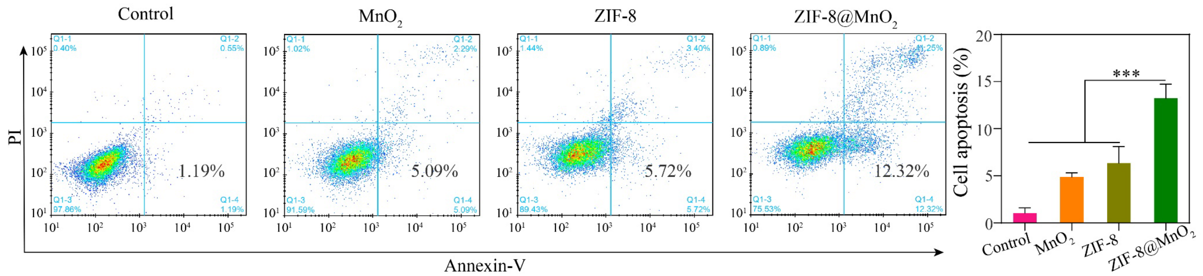

Figure S15. Flow cytometry detection of apoptosis in BT549 cells treated with PBS, MnO_2, ZIF-8, and ZIF-8@MnO_2 (n = 3, mean ± SD). One-way ANOVA analyses were performed: ***$p < 0.001$.

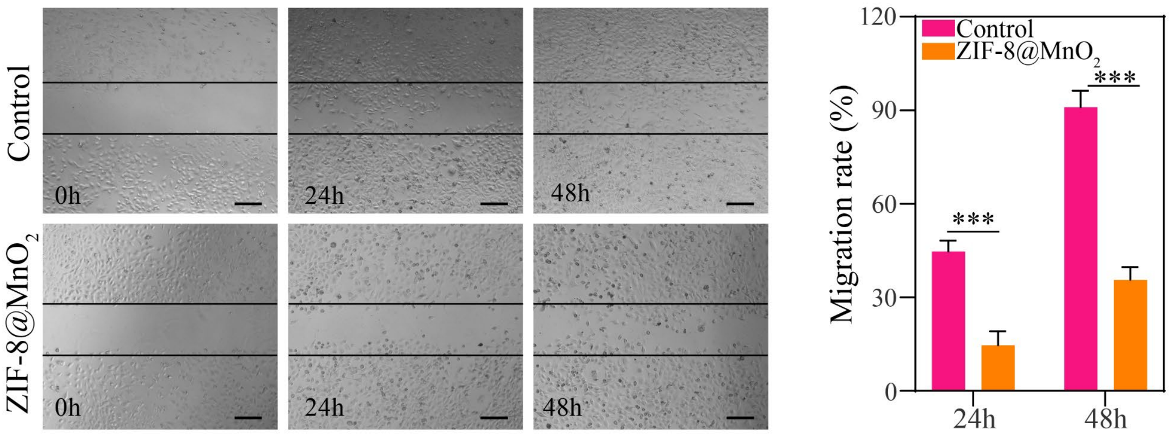

Figure S16. Migration ability of BT549 cells after ZIF-8@MnO_2 treatment compared to PBS (n = 3, mean ± SD). Scale bar: 100 μm. Statistical analysis was calculated by Student's t-test: ***$p < 0.001$.

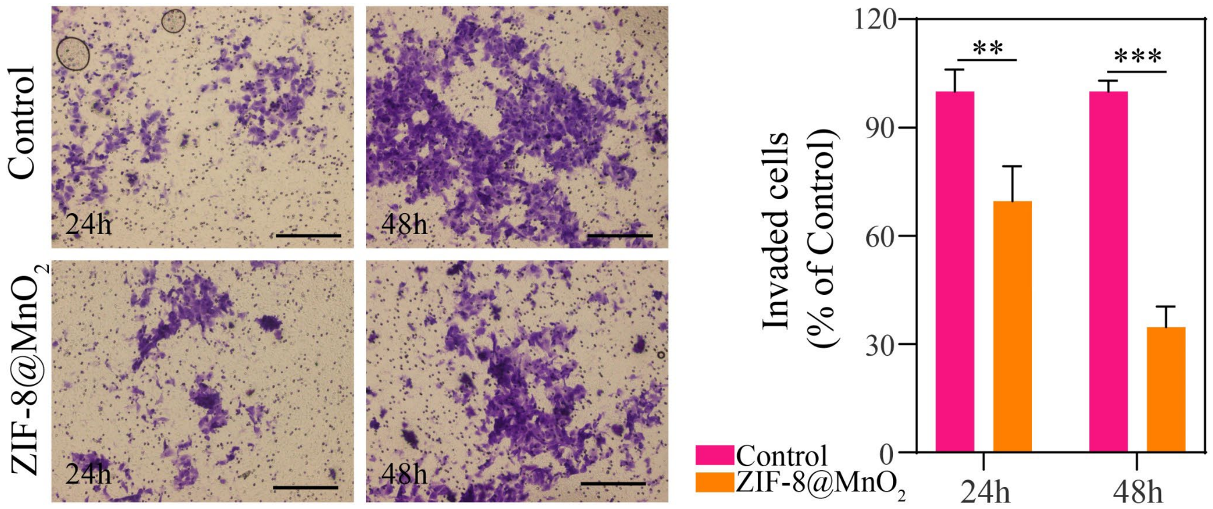

Figure S17. Invasive ability of BT549 cells after ZIF-8@MnO_2 treatment compared to PBS. Scale bar: 100 μm. Statistical analysis was calculated by Student's t-test: **p < 0.01, ***p < 0.001.

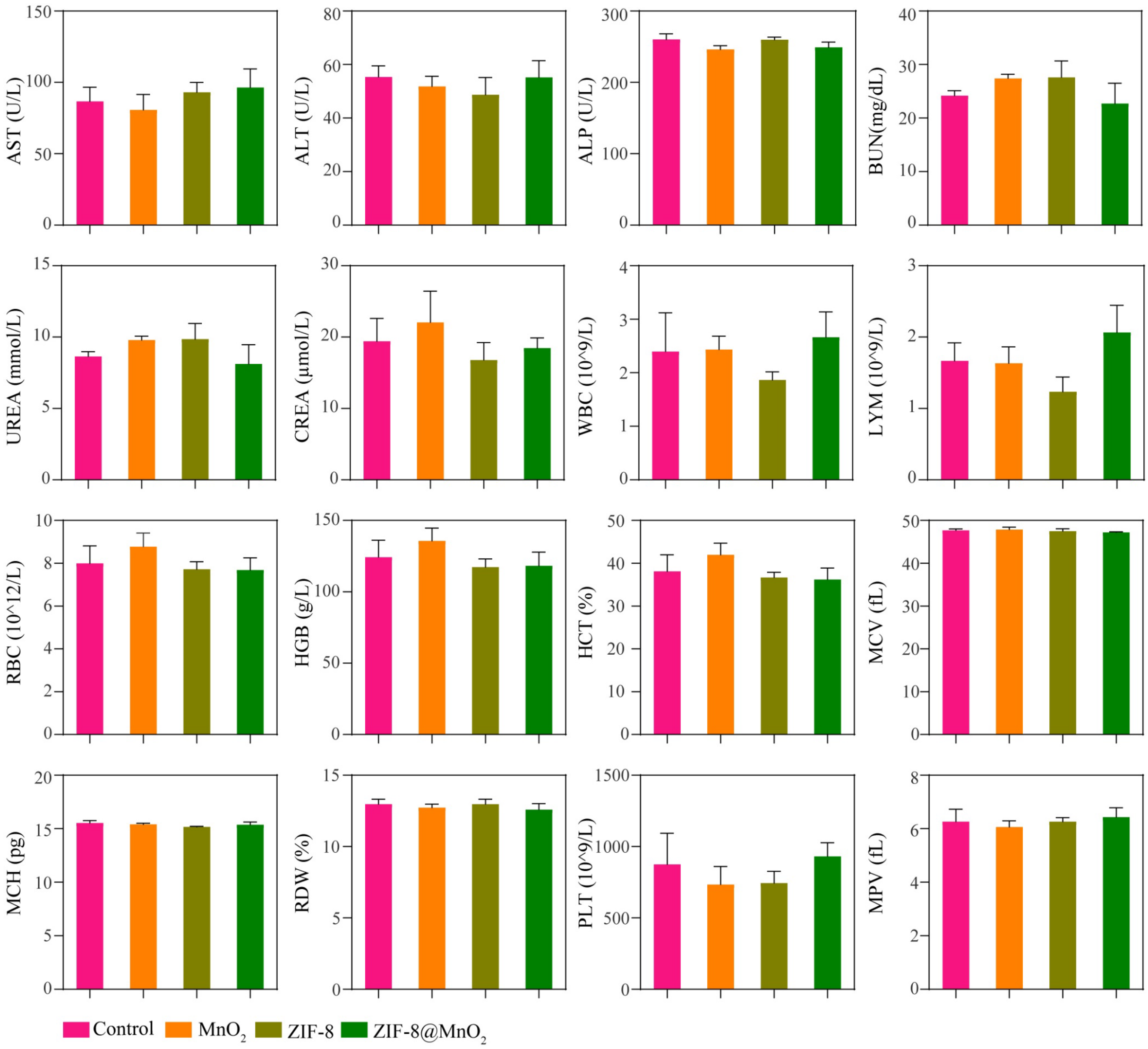

Figure S18. Routine blood test and biochemical evaluation of the BALB/c mice after different treatments (PBS, MnO_2, ZIF-8, ZIF-8@MnO_2) (Zn, 6 mg/kg) on the 15th day. No difference was found between several groups, indicating good biosafety of the nanoparticles *in vivo*.

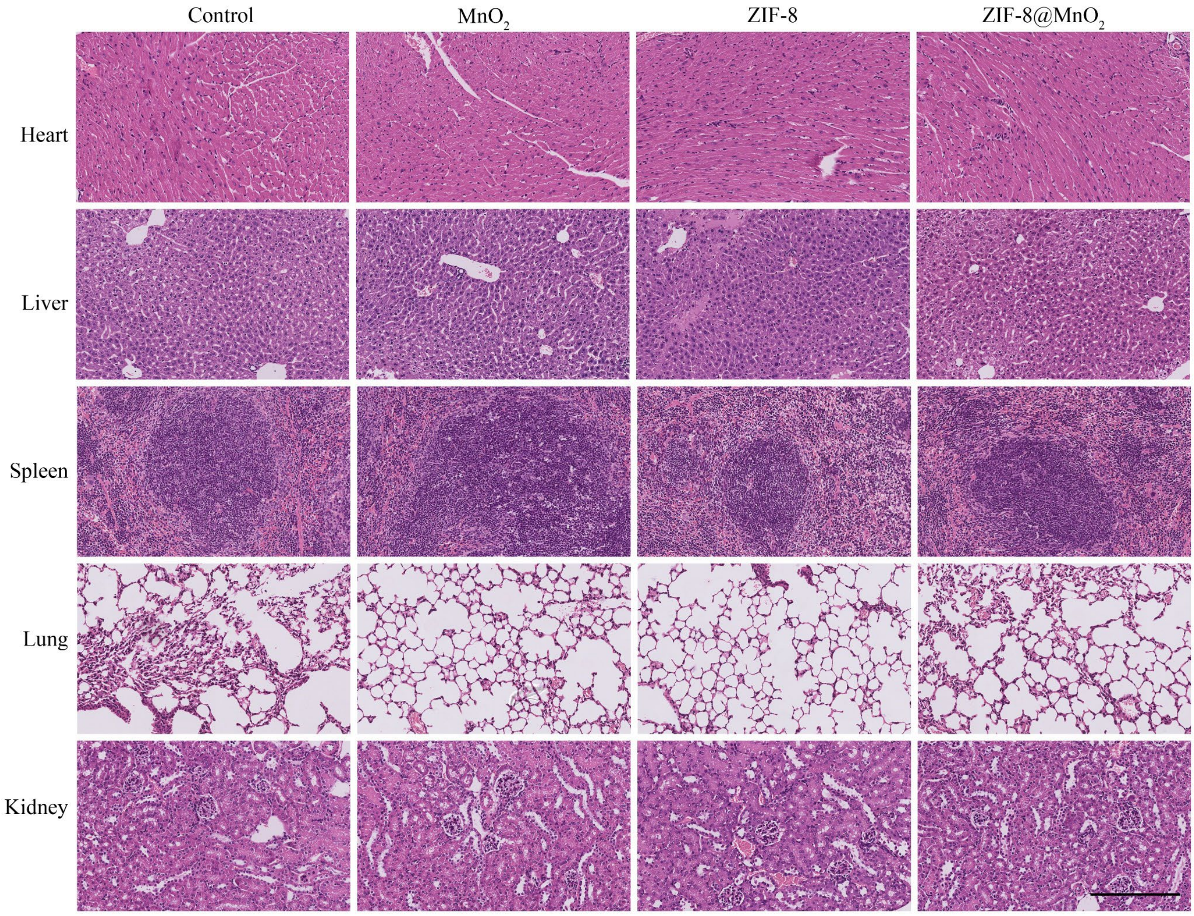

Figure S19. H&E staining was performed to evaluate pathological changes in the heart, liver, spleen, lungs, and kidneys of the mice treated with PBS, MnO_2, ZIF-8, and ZIF-8@MnO_2 (Zn, 6 mg/kg) on the 15th day, showing no significant change of H&E tissue sections. Scale bar = 100 μm.

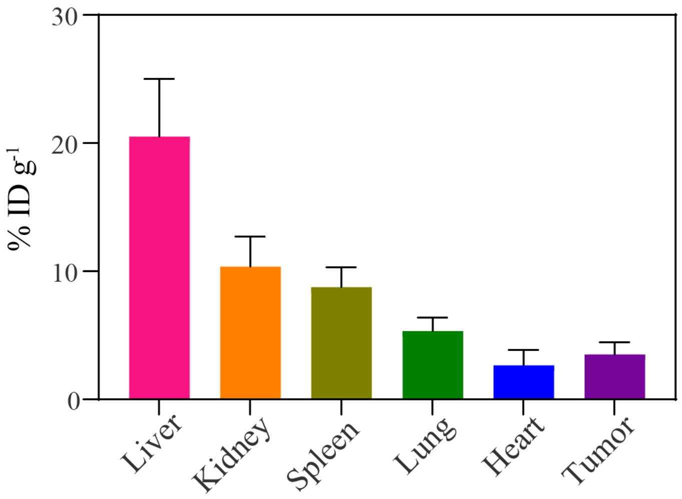

Figure S20. Biodistribution of the ZIF-8@MnO_2 nanoparticles in tumor-bearing mice after *i.v.* injection of the nanoparticles (Zn, 6 mg/kg) on the 1st day.

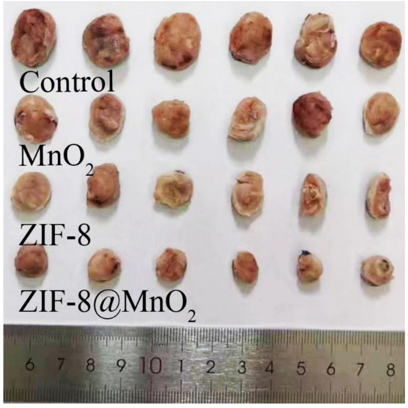

Figure S21. Photo of tumors harvested on the 15th day post-treatment.

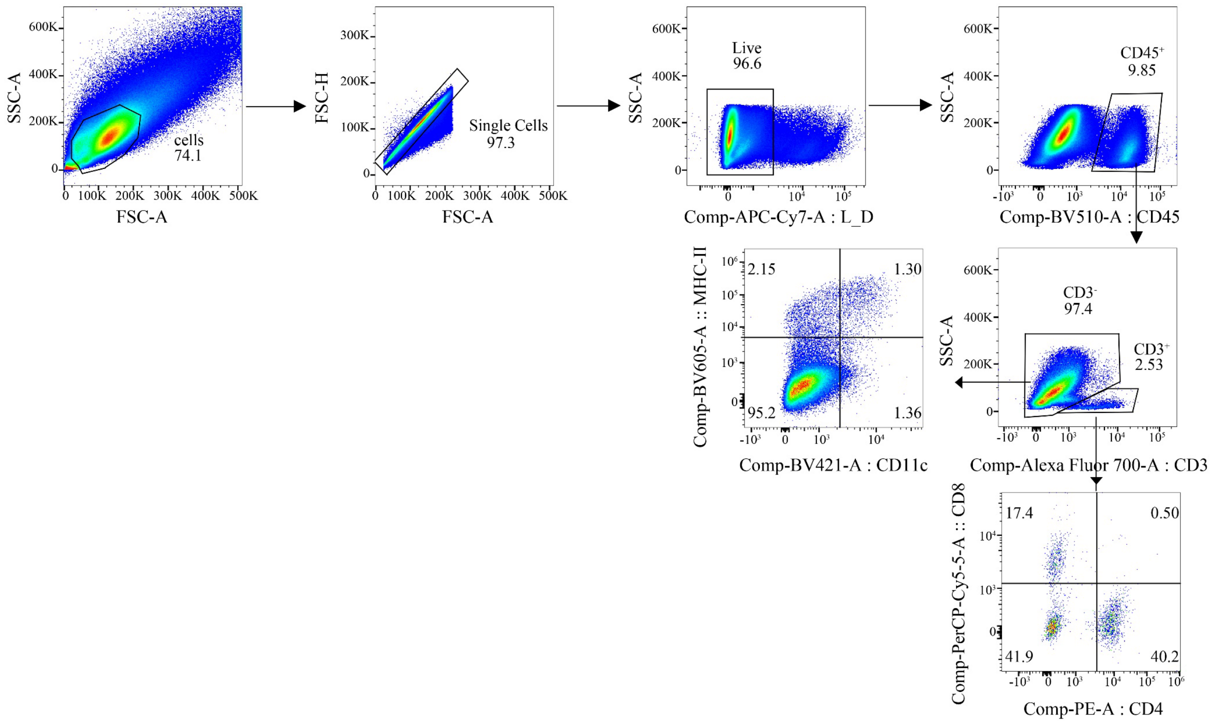

Figure S22. Flow cytometry gating strategy for the analysis of DC and CD3$^+$ CD8$^+$, CD3$^+$ CD4$^+$ T cells *in vivo*.

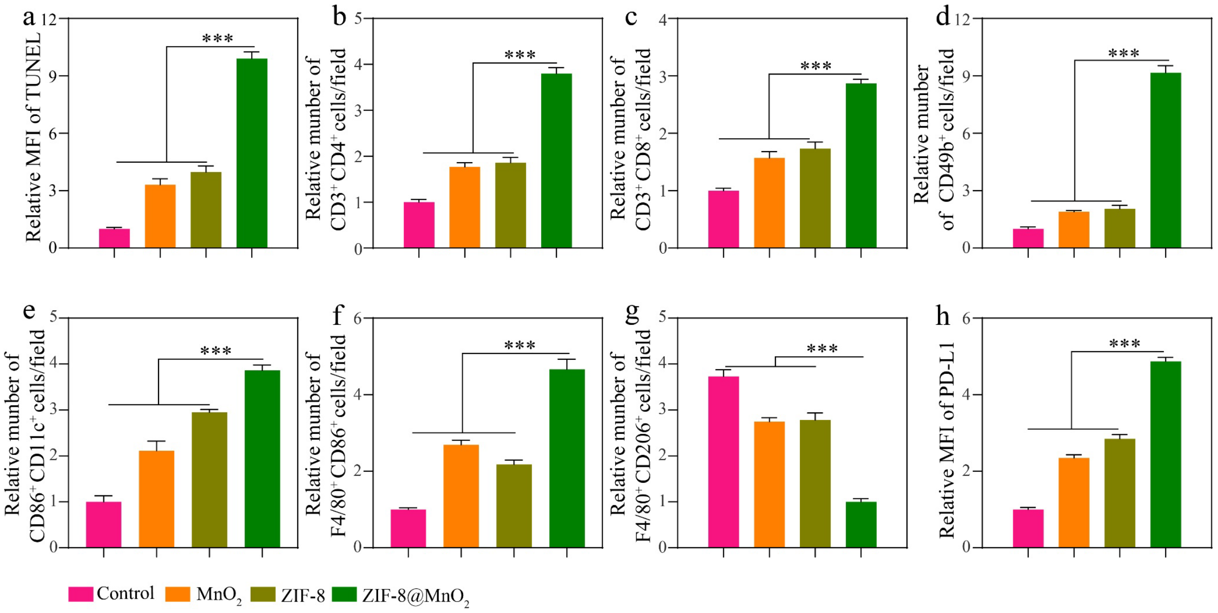

Figure S23. Statistical analysis of immuno-fluorescence staining of a) TUNEL, b) $CD3^+ CD4^+$, c) $CD3^+ CD8^+$, d) $CD49b^+$, e) $CD86^+ CD11c^+$, f) F4/$80^+ CD86^+$, g) F4/$80^+ CD206^+$, h) PD-L1 in tumor tissues of each group. One-way ANOVA analyses were performed: ***$p < 0.001$.

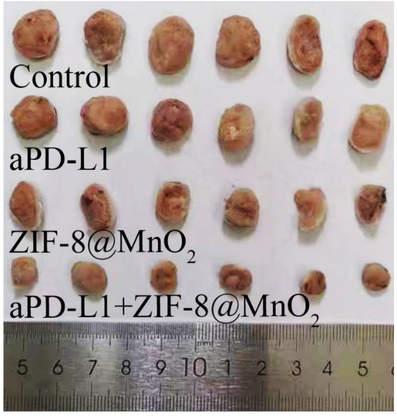

Figure S24. Photo of tumors harvested on the 15th day post-treatment with PD-L1 blocking antibody.

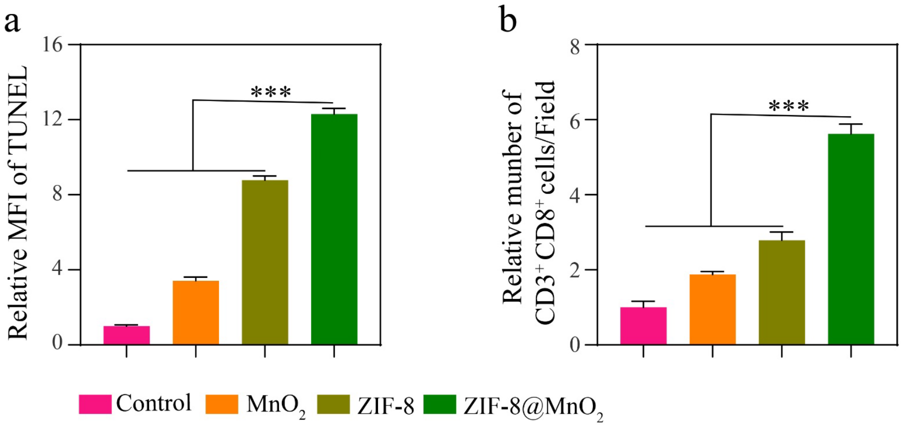

Figure S25. Statistical analysis of immuno-fluorescence staining of a) TUNEL and b) CD3$^+$ CD8$^+$. One-way ANOVA analyses were performed: ***$p < 0.001$.

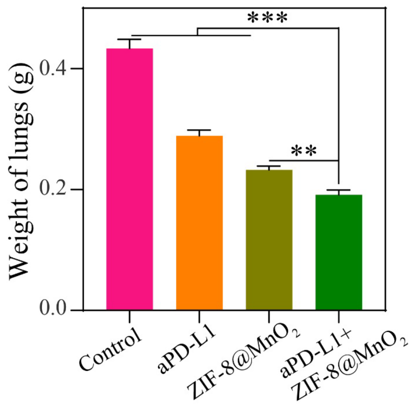

Figure S26. Lung weight of lung metastatic 4T1 tumor models after different treatments. One-way ANOVA analyses were performed: **p < 0.01, ***p < 0.001.

References

[1] B. Ding, H. Chen, J. Tan, Q. Meng, P. Zheng, P. Ma, J. Lin, *Angew. Chem. Int. Ed. Engl.* **2023**, 62, e202215307.
[2] Z. Chen, L. Chen, Y. Ma, Y. Liu, Q. Zhang, H. Qin, Y. Chen, B. Tian, J. Dong, *Adv. Mater.* **2023**, e2304246.
[3] X. Liu, K. Yin, L. Chen, W. Chen, W. Li, T. Zhang, Y. Sun, M. Yuan, H. Wang, Y. Song, S. Wang, S. Hu, Z. Zhou, *Cell Discov* **2023**, 9, 6.
[4] R. Song, S. Ma, J. Xu, X. Ren, P. Guo, H. Liu, P. Li, F. Yin, M. Liu, Q. Wang, L. Yu, J. Liu, B. Duan, N. A. Rahman, S. Wołczyński, G. Li, X. Li, *Mol. Cancer* **2023**, 22, 16.
[5] R. Wang, A. B. Bhatt, B. A. Minden-Birkenmaier, O. K. Travis, S. Tiwari, H. Jia, W. Rosikiewicz, O. Martinot, E. Childs, R. Loesch, G. Tossou, S. Jamieson, D. Finkelstein, B. Xu, M. Labelle, *Sci Adv* **2023**, 9, eabq3951.
